# Genetic analysis of ocular tumour-associated genes using large genomic datasets: insights into selection constraints and variant representation in the population

**DOI:** 10.1136/bmjophth-2023-001565

**Published:** 2024-02-21

**Authors:** Alexander Tanner, Mandeep S Sagoo, Omar A Mahroo, Jose S Pulido

**Affiliations:** 1 Institute of Ophthalmology, University College London, London, UK; 2 Retinal Genetics Service, Moorfields Eye Hospital NHS Foundation Trust, London, UK; 3 Retinoblastoma Service, The Royal London Hospital, London, UK; 4 Oncology Service, Moorfields Eye Hospital NHS Foundation Trust, London, UK; 5 Wills Eye Hospital, Philadelphia, Pennsylvania, USA

**Keywords:** Genetics, Neoplasia

## Abstract

**Background:**

Large genomic databases enable genetic evaluation in terms of haploinsufficiency and prevalence of missense and synonymous variants. We explored these parameters in ocular tumour-associated genes.

**Methods:**

A curated list of ocular tumour-associated genes was assessed using the genomic databases Genome Aggregation Database (gnomAD) and DatabasE of genomiC varIation and Phenotype in Humans using Ensembl Resources (DECIPHER) and compared with breast and lung cancer-associated gene lists. Haploinsufficiency was determined based on specific criteria: probability of loss of function index ≥0.9 in gnomAD, upper CI O/E limit <0.35 for loss of function variants in gnomAD and/or a DECIPHER pHaplo ≥0.86. UniProt was used for further gene characterisation, and gene ontology Protein Analysis THrough Evolutionary Relationships was explored for common biological pathways. In addition, we identified genes with under-representation/over-representation of missense/synonymous variants.

**Results:**

Fifty-seven genes were identified in association with ocular and extraocular tumours.

Regarding haploinsufficiency, 41% of genes met the criteria for negative selection, with 57% categorised as tumour-suppressing and 39% as oncogenic. Most genes were involved in regulatory processes. Regarding triplosensitivity, 33% of genes reached significance and 83% of these were haploinsufficient. Analysis of variants revealed under-representation of missense variants in 23% of genes and over-representation of synonymous variants in 5% of genes. Ocular tumour-associated genes exhibited higher scores for haploinsufficiency and triplosensitivity compared with breast and lung cancer-associated genes. Pathway analysis revealed significant enrichment in cellular proliferation, differentiation and division. Encoded proteins of ocular tumour-associated genes were generally longer than the median of the UniProt database.

**Conclusion:**

Our findings highlight the importance of negative selection in ocular tumour genes, supporting cranial gene conservation. This study provides insights into ocular tumourigenesis and future research avenues.

**WHAT IS ALREADY KNOWN ON THIS TOPIC**

- Large genomic databases enable the evaluation of genes based on parameters such as haploinsufficiency, triplosensitivity and the prevalence of missense or synonymous variants.

**WHAT THIS STUDY ADDS**

- Using the above parameters for ocular tumour-associated genes: 23 genes were identified as haploinsufficient, 18 were triplosensitive, 13 showed under-representation of missense variants and 3 showed over-representation of synonymous variants.

**HOW THIS STUDY MIGHT AFFECT RESEARCH, PRACTICE OR POLICY**

- These findings highlight the importance of negative selection in ocular tumourigenesis and can be used for comparison with future genetic studies.

## Background

Ocular tumours can manifest either as local, somatic mutations or as germline mutations in individuals with hereditary tumour predisposition syndromes.^1^ Among these tumours are retinoblastoma and uveal melanoma, both of which are rare but can have devastating consequences. Retinoblastoma, a paediatric neoplasm, is the most prevalent primary intraocular tumour worldwide, with an estimated incidence of 7202–8102 children each year.^2^ It arises from cells that harbour cancer-associated variants in both copies of their *RB1* genes. This can be inherited in an autosomal dominant pattern or can occur spontaneously. Despite advancements in diagnosis and treatment, the mortality rate for retinoblastoma remains high at 70% in low-income and middle-income countries.^3^ In adults, uveal melanoma is the leading primary malignancy affecting the eye, impacting an estimated 7000 individuals worldwide each year.^4^ *GNAQ* and *GNA11* are the most frequently mutated genes in uveal melanoma, with mutations occurring in 71%–93% of associated tumours.^5^ Notably, the risk of treatment-resistant metastatic disease contributes to persistently high mortality rates, with some studies reporting long-term mortality rates exceeding 50% for this condition.^6^

Genetic studies enhance our understanding of the disease pathways underpinning ocular tumours. Moreover, the emergence of large genomic databases has facilitated the evaluation of genes based on parameters such as intolerance to loss of function (‘haploinsufficiency’) and the prevalence of missense or synonymous variants. Haploinsufficiency refers to a genetic condition wherein the presence of only one functional copy of a specific gene in a diploid organism is insufficient to maintain normal cellular function. In this context, the remaining single functional copy of the gene is incapable of producing the level of gene product required for proper biological functioning. This may lead to various developmental abnormalities, increased susceptibility to diseases or other medical conditions, depending on the specific gene and its role in cellular processes.

We used two databases, namely ‘The Genome Aggregation Database’ (gnomAD)^7^ and ‘DatabasE of genomiC varIation and Phenotype in Humans using Ensembl Resources’ (DECIPHER),^8^ to investigate these parameters in genes associated with ocular tumours. gnomAD incorporates data from over 141 456 individuals sequenced with 125 748 exomes and 15 708 genomes, aligned against the Genome Reference Consortium Human genome build 37.^7^ DECIPHER, another extensive database, contains genomic data from 33 000 children with rare diseases from 250 centres.^8^ By analysing these databases, we aimed to determine if the genes implicated in ocular tumours exhibit selection constraints and whether their variants are over-represented or underrepresented in the population.

## Methods

### Overview

We used a similar, but updated methodology to our previously published investigation of inherited retinal disease (IRD)-associated genes.^9^

A list of intraocular and extraocular tumours, and their related genes, was generated using MalaCards: The human disease database (https://www.malacards.org/).^10^ This was supplemented by two ocular oncologists querying the Online Mendelian Inheritance in Man (OMIM) genetic database (https://omim.org/about)^11^ and by performing a systematic search for articles that listed pseudomelanomas, pseudogliomas and orbital tumours in PubMed.^12 13^ This curation of tumours and related genes was performed in January 2021 and was updated in June 2023.

Evaluation of these genes for haploinsufficiency, triplosensitivity and the degree of missense and synonymous variation was conducted using the online databases (gnomAD and DECIPHER). This analysis was originally performed in January 2021 and was updated in June 2023.

### Genome Aggregation Database

The constraint variables in gnomAD (https://gnomad.broadinstitute.org/) include pLI (probability of loss of function intolerance), O (observed, which is the frequency of the particular variant in the database), E (expected which is the expected frequency in the database assuming that the variant develops randomly), O/E (observed divided by expected, which is the ratio of observed variants to expected) and CI (the CI for the O/E). The pLI ranges from 0 to 1, and a pLI of 0.9 or greater is a strong indicator that loss of function variants in the gene are selected against. This is confirmed when the upper CI for loss of function is 0.35 or less.^7^ The O/E for missense and synonymous variants can also be explored: for this study Z scores of 2.99 or greater, or −2.99 or less, were taken to indicate a significant over-representation or under-representation of missense and synonymous variants (a Z value of −2.99 means that the chance of variants occurring randomly with such low frequency in the population is only 0.14% (0.0014)).

### DatabasE of genomiC varIation and Phenotype in Humans using Ensembl Resources

DECIPHER (https://decipher.sanger.ac.uk) comprises genomic data from 36 000 children with rare diseases from over 270 specialist centres.^8^ Previously, a haploinsufficiency score was provided, where an index of less than 10% was taken to indicate that loss of function is significantly selected against.^14^ This has now been replaced by the updated ‘pHaplo’ and ‘pTriplo’ scores which enable the evaluation of haploinsufficiency and triplosensitivity, respectively.^8^ To ensure greater accuracy, a pHaplo of ≥0.86 and a pTriplo of ≥0.94 were adopted as per Collins *et al*.^15^

### Combined selection criteria

Those ocular cancer genes that met the following criteria for haploinsufficiency: (1) a pLI in gnomAD of ≥0.9 and (2) an upper CI O/E limit for loss of function variants in gnomAD of <0.35 and/or a DECIPHER pHaplo of ≥0.86 were then further characterised, using UniProt and evaluated for the presence of common biological pathways using the online gene ontology resource gene ontology Protein Analysis THrough Evolutionary Relationships (GO PANTHER).^16^

### Protein Analysis THrough Evolutionary Relationships

Identification of common biological pathways was achieved by inputting our list of haploinsufficient genes through the gene ontology PANTHER (http://pantherdb.org/) resource.^16^

### UniProt

Amino acid lengths of the encoded proteins of all identified genes were obtained from the UniProt Database (www.uniprot.org), a comprehensive resource that details protein sequence and functional information.^17^

### Comparisons

This list of ocular tumour genes was then compared with two lists of genes generated by MalaCards and supplemented by a comprehensive search of OMIM and PubMed articles, one associated with breast cancer and the other with lung cancer.^10 11 18^ The analysis was performed using gnomAD, DECIPHER, PANTHER and UniProt.^8 11 16 17^

## Results

### Ocular tumour-associated genes

There were 57 genes identified in association with ocular and extraocular tumours (online supplemental table 1). For 56 of the ocular tumour-associated genes, excluding *DUX4* due to insufficient data, the median of the haploinsufficiency variables was as follows: pLI of 0.72, O/E of 0.19 and pHaplo 0.89. Of these 56 genes, 23/56 (41%) met our combined criteria for negative selection when haploinsufficient (table 1): (1) A pLI in gnomAD of ≥0.9 and (2) an upper CI O/E limit for loss of function variants in gnomAD of <0.35 and/or a DECIPHER pHaplo of ≥0.86.

10.1136/bmjophth-2023-001565.supp1 Supplementary data

**Table 1:** Table depicting the haploinsufficient genes identified in ocular tumour-associated disease, breast cancer and lung cancer

Ocular tumour-associated genes	Breast cancer-associated genes	Lung cancer-associated genes
APC	AKT1	ABL1
BAP1	ESR1	AKT1
BRAF	MSH2	ARID1A
CDK6	PIK3CA	ARID2
CTNNB1	RET	BRAF
DICER1	STK11	CTNNB1
GNAQ		CTNND2
KIF11		FBXW7
MDM2		FGF10
MITF		GNAQ
MYC		IRF1
MSH2		KMT2C
NF1		KMT2D
NF2		MAP2K1
PLAG1		MAPK1
PTCH1		MET
RB1		PIK3CA
SF3B1		PPP2R1A
SUFU		RB1
TERT		RELN
TSC1		REV3L
TSC2		
U2AF1		
		SLC25A5
		SMARCA4
		STK11
		TERT
		ZEB2
		ZFHX3
		ZNF521

Among these 23 haploinsufficient genes, 57% (13/23) were identified as tumour-suppressing, while 39% (9/23) exhibited oncogenic properties. Furthermore, most of these genes, 87% (20/23), were found to be involved in regulatory processes. A smaller subset, 9% (2/23), was associated with DNA repair, and 4% (1/23) were identified as mechanochemical.

Regarding triplosensitivity, the median score for these genes was 0.71 (pTriplo). Out of the 54 genes analysed, 33% (18/54) reached significance (pTriplo≥0.94), with exclusion of *EIF1AX* and *NDP* in addition to *DUX4,* due to insufficient data. 83% (15/18) of the triplosensitive genes also met our criteria for haploinsufficiency.

Thirteen of the 56 genes (23%) demonstrated under-representation of missense variants (Z score ≥2.99), of which 10/13 (77%) also met our criteria for haploinsufficiency. The median missense score of the 56 genes was 1.48 (Z score). No ocular tumour-associated genes demonstrated over-representation of missense variants. Three out of 56 genes (5%) demonstrated over-representation of synonymous variants: *MC1R*, *PTCH1* and *TSC2*. Incidentally, *PTCH1* and *TSC2* were also found to meet our criteria for haploinsufficiency. No ocular tumour-associated genes demonstrated under-representation of synonymous variants. The median synonymous score of the 56 genes was −0.08 (Z score).

### Breast cancer-associated genes

A total of 29 breast cancer-associated genes were identified. Among these genes, 6 out of 29 (21%) met the study’s criteria for haploinsufficiency (table 1). For these 29 breast cancer genes, the median of the haploinsufficiency variables was pLI 0, O/E 0.60 and pHaplo 0.68. Regarding the haploinsufficient breast cancer-associated genes, 50% of the genes were identified as tumour suppressing, while the remaining 50% were classified as oncogenic. Additionally, 83% (5/6) of the genes were found to be involved in regulatory processes and 17% (1/6) were associated with DNA repair.

Out of the 29 genes analysed, 5 (17%) met the criteria for triplosensitivity, and the median triplosensitivity score for the breast cancer-associated genes was 0.43 (pTriplo). Out of the five triplosensitive genes identified, *AKT1* and *PIK3CA* (40%) also met our criteria for haploinsufficiency.

Regarding missense variants, 3/29 (10%) of the genes (*AKT1, PIK3CA* and *PTEN*) exhibited significantly fewer variants than would be expected. The median missense score of the 29 genes was 0.58 (Z score). No breast cancer genes demonstrated over-representation of missense variants. However, one genes, *MSH6*, demonstrated over-representation of synonymous variants. None of the breast cancer-associated genes showed under-representation of synonymous variants. The median synonymous score for the 29 genes was −0.13 (Z score).

### Lung cancer-associated genes

Seventy-five lung cancer-associated genes were identified. Among these, 28/75 (37%) were observed to be negatively selected for when haploinsufficient (table 1). The median values of the haploinsufficiency variables for lung cancer were as follows: pLI 0.26, O/E 0.24 and pHaplo 0.78. Out of those haploinsufficient lung cancer-associated genes, 43% (12/28) were identified as oncogenic, while 46% (13/28) were categorised as tumour suppressing. 79% (22/28) of the genes were found to be involved in regulatory processes, 4% (1/28) in DNA repair and 11% (3/28) mechanochemical.

Twenty-five out of the 75 lung cancer-associated genes (33%) were found to meet our criteria for triplosensitivity and the median triplosensitivity score was 0.75 (pTriplo). 72% (18/25) of the triplosensitive genes also met our criteria for haploinsufficiency.

Sixteen of the 75 lung cancer-associated genes (21%) exhibited under-representation of missense variants. Additionally, two genes (MUC4 and MUC16) demonstrated over-representation of missense variants. Six out of the 75 lung cancer-associated genes were found to demonstrate under-representation of synonymous variants (8%). No genes showed over-representation of synonymous variants. The median synonymous score for the 75 genes was −0.52 (Z score).

### Protein Analysis THrough Evolutionary Relationships

When performing PANTHER analysis on the 23/56, ocular tumour-associated, haploinsufficient genes >100 fold enrichment in 14 biological pathways were observed including, cellular proliferation, differentiation and division (online supplemental table 2).

10.1136/bmjophth-2023-001565.supp2 Supplementary data

PANTHER analysis of the 6/29 haploinsufficient breast cancer-associated genes and 28/75 lung cancer-associated genes demonstrated >100 fold enrichment in 33 (online supplemental table 3) and 15 (online supplemental table 4) biological pathways, respectively.

10.1136/bmjophth-2023-001565.supp3 Supplementary data

10.1136/bmjophth-2023-001565.supp4 Supplementary data

### UniProt

Most encoded proteins were longer than the median length in the UniProt database.^19^ The median amino acid length of the ocular tumour-associated genes was 605 (781 for the haploinsufficient group), 754 for breast cancer genes (764.5 for the haploinsufficient group) and 781 for lung cancer genes (1099 for the haploinsufficient group). While the median amino acid lengths for triplosensitive ocular tumour-associated genes, breast cancer-associated genes and lung cancer-associated genes were 667, 480 and 824, respectively.

## Discussion

Many ocular tumour-associated-genes, when haploinsufficient, are strongly associated with negative selection. In contrast, we have previously shown that for IRDs, relatively few genes were associated with negative selection for loss of function variants.^9^ It should be noted that many of these ocular tumour-associated-genes develop their mutations as somatic mutations. This is consistent with the fact that genetic mutations are negatively selected for since they are involved in important cellular pathways that would affect the development of many organs in the body. Recently, it has been shown that in certain cells there is a temporal spectrum for mutations to occur, from early in the germline until late into adulthood.^20^

Compared with breast and lung cancer-associated genes, the ocular tumour-associated genes identified in our study exhibited higher scores for haploinsufficiency. Moreover, there was an equal proportion (compared with lung cancer) and greater proportion (compared with breast cancer) of negatively selected haploinsufficient ocular tumour-associated genes. This finding highlights the inherent importance of many cranial-associated genes and supports the notion that these tissues are evolutionarily conserved compared with other somatic tissues.^21 22^

In an attempt to characterise these haploinsufficient genes, we have found that most of them encode proteins involved in regulatory processes. This was the same for ocular tumour-associated, breast cancer-associated and lung cancer-associated genes. This supports the notion that these encoded proteins are likely to be ‘structural’, ‘regulatory ‘and/or ’mechanochemical’.^23^ Moreover, our data present a similar finding to Niemitz, with a large proportion of haploinsufficient genes acting as tumour suppressors and a large proportion of triplosensitive genes acting as oncogenes.^24^

Collins *et al* demonstrated some interesting findings in relation to haploinsufficient genes.^15^ These genes tended to be larger in size, located farther away from other genes and possessed a greater number of conserved enhancers in cis. These characteristics are considered classic indicators of precisely regulated, developmentally critical genes. On the other hand, triplosensitive genes were generally shorter, rich in G/C content and located in gene-dense, highly active regions, which were not particularly enriched for conserved enhancers.^15^ Our data also show that the median size of the haploinsufficient genes is larger than the median of all the identified genes, per category. Additionally, the median size of the triplosensitive genes is smaller than that of the haploinsufficient genes, per category, but larger than the median size of all the identified genes, except for breast cancer-associated genes. It should be noted that pLI scores, for a given combination of selection parameters, can vary greatly with gene length.^25^

There are several limitations to this study that require mentioning. It is likely that there are other genes involved in the development of breast cancer, lung cancer and ocular tumours that this study has missed or that have not yet been discovered. Moreover, the gene lists include tumour suppressor genes, regulatory genes and oncogenes responsible for somatic tumours, as well as rare severe Mendelian disorders, some of which are lethal in the homozygous state. It should also be mentioned that the determinant of ‘haploinsufficiency’ that we use relies on scores derived from population genetics, such as ‘pLI’. These scores in fact reflect the strength of selection on heterozygotes and are not direct indicators of haploinsufficiency relative to phenotype,^25^ making it challenging to draw firm conclusions from the data. The observed difference between our results in this paper and that of our previous paper on IRDs may reflect the variance in disease types and, consequently, differing selection pressures. It is also important to highlight that the interpretation of our findings may have been influenced by the cross-over of certain genes across the analysed categories: ocular tumour-associated, breast cancer-associated and lung cancer-associated genes. Nonetheless, this study lays a foundation for evaluating genes by a novel and potentially useful means. Additionally, a very stringent level of a Z value of ≤−2.99 or ≥2.99 was used that may have resulted in some genes being omitted from the study that would otherwise have been included, should the Z value have been less stringent. Finally, we included genes that are associated with intraocular, orbital and pseudogliomas in this study.

## Data Availability

All data relevant to the study are included in the article or uploaded as online supplemental information.
