# Supplementary material for: Genetic analysis of ocular tumour-associated genes using large genomic datasets: insights into selection constraints and variant representation in the population
Source: BMJ Open Ophthalmol. 2024 Feb 21;9(1):e001565. doi: 10.1136/bmjophth-2023-001565 (PMC10897839; doi:10.1136/bmjophth-2023-001565)
Supplement: Supplementary data [file bmjophth-2023-001565supp001.pdf]

**Supplementary Table 1:** Table depicting the ocular tumour-associated genes, their location, function, and associated diseases.

| Gene: | Location: | Function: | Disease: |
|---|---|---|---|
| ABCG2 | 4q22.1 | ATP Binding Cassette Subfamily G Member 2 | Retinoblastoma |
| APC | 5q22.2 | Tumour suppressor gene.<br>Regulator of Wnt Signalling Pathway. | Congenital hypertrophy of retinal pigment epithelium, Gardner syndrome, ocular surface squamous cell neoplasia. |
| BAP1 | 3p21.1 | Tumour suppressor gene.<br>A deubiquitylase, thought to be a key regulator of cell cycle, growth, response to DNA damage and chromatin dynamics. | Uveal melanoma, conjunctival melanoma |
| BRAF | 7q34 | Proto-oncogene. Raf family of serine/threonine kinases. Key regulator MAP kinase/ ERK signalling pathway, affecting cell division, differentiation, and secretion. | Conjunctival melanoma, cutaneous melanoma, uveal melanoma |
| BRCA2 | 13q13.1 | Tumour suppressor gene. DNA Repair Associated. | Uveal melanoma |
| CCND1 | 11q13.3 | Proto-oncogene. Regulatory protein in G1/S transition of cell cycle. | Von Hippel-Lindau Syndrome, Uveal melanoma |
| CDK4 | 12q14.1 | Proto-oncogene. Serine/threonine kinase involved in cell cycle regulation and repair of damage caused by UV radiation. | Uveal melanoma, conjunctival melanoma |
| CDK6 | 7q21.2 | Proto-oncogene. Serine/threonine kinase involved in cell cycle and differentiation; promotes G1/S transition. | Uveal melanoma, conjunctival melanoma |

| | | | |
|---|---|---|---|
| CDKN2A | 9p21.3 | Tumour suppressor gene. Capable of inducing cell cycle arrest in G1 and G2 phases. | Uveal melanoma, neurofibromatosis |
| CTC1 | 17p13.1 | Putative tumour suppressor gene. Component *CST* complex believed to play a role in promoting DNA replication. | Cerebroretinal microangiopathy with calcifications and cysts |
| CTNNB1 | 3p22.1 | Proto-oncogene. Key downstream component of canonical Wnt signalling pathway. | Teratocarcinosarcoma of the eye |
| CYSLTR2 | 13q14.2 | Proto-oncogene. Involved in the production and activity of inflammatory mediators. | Uveal melanoma |
| DICER1 | 14q32.13 | Haploinsufficient tumour suppressor gene with loss of one allele leading to tumour progression but loss of both alleles having an inhibitory effect on tumour development. Endoribonuclease involved in post-transcriptional gene silencing. | Ciliary body medulloepithelioma, rhabdomyosarcoma |
| DMPK | 19q13.32 | Mediator of tumour suppressor genes. Various functions including maintenance of skeletal/ cardiac muscle structure and function, synaptic plasticity and regulation of chloride currents. | Uveal melanoma |
| DUX4 | 4q35.2 | Tumour suppressor/ proto-oncogene. Transcription factor involved in embryogenesis, physiology & disease. | Coats-like syndrome, exudative retinopathy |
| EIF1AX | Xp22.12 | Proto-oncogene. Encodes an essential eukaryotic | Uveal melanoma |

| | | | |
|---|---|---|---|
| | | translation initiation factor. Required for binding of *43S* complex to the 5' end of capped RNA. | |
| ERCC2 | 19q13.32 | DNA repair gene involved in separating the double helix via the 5'-3' helicase activity. | Xeroderma pigmentosum, ocular squamous cell carcinoma |
| FHIT | 3p14.2 | Tumour suppressor gene. Important in regulation of apoptosis. Involved in purine metabolism. | Uveal melanoma |
| FLCN | 17p11.2 | Tumour suppressor gene. Multi-functional protein, involved in both the cellular response to amino acid availability and in the regulation of glycolysis. | Birt-Hogg-Dubé syndrome, choroidal melanoma, uveal melanoma, lid folliculoma |
| GNA11 | 19p13.3 | Proto-oncogene. G Protein Subunit Alpha 11 | Uveal melanoma, choroidal |
| GNAQ | 9q21.2 | Proto-oncogene. G Protein Subunit Alpha Q | Sturge weber syndrome, uveal melanoma, nevus of ota |
| KDM4C | 9p24.1 | Regulator of oncogenes and tumour suppressor genes. Nuclear protein involved in histone modification and a regulator of several transcription factors. | Uveal melanoma |
| KIF11 | 10q23.33 | Proto-oncogene. Protein involved in spindle dynamics during mitosis. Also involved in retinal vascular development. | Familial exudative vitreoretinopathy |
| LRP5 | 11q13.2 | This gene encodes a transmembrane low-density lipoprotein receptor that binds and internalises ligands in the process of receptor-mediated endocytosis | Osteoporosis-pseudoglioma syndrome |

| | | | |
|---|---|---|---|
| MAPKAPK5 | 12q24.12-q24.13 | Tumour suppressor gene. Serine/threonine kinase involved in cellular stress and inflammatory response. | Uveal melanoma |
| MC1R | 16q24.3 | Tumour suppressor gene. Encodes melanocortin one receptor. | Uveal melanoma |
| MDM2 | 12q15 | Important negative regulator of *p53* tumour suppressor. | Uveal melanoma, retinoblastoma |
| MITF | 3p13 | Tumour suppressor gene. Regulates differentiation and development of melanocytes and retinal pigment epithelium. | Uveal melanoma |
| MLANA | 9p24.1 | Protein antigen found on the surface of melanocytes. | Uveal melanoma, conjunctival melanoma, malignant spindle cell melanoma |
| MLH1 | 3p22.2 | Tumour suppressor gene. Involved in post-replicative DNA mismatch repair. | Keratocanthoma, Muir-Torre syndrome, sebaceous adenoma, cutaneous melanoma |
| MYC | 8p24.21 | Proto-oncogene. Multifunctional, nuclear phosphoprotein involved in cell cycle, cell growth, apoptosis, metabolism, biosynthesis, adhesion, and mitochondrial biogenesis. | Uveal melanoma |
| MSH2 | 2p21-p16 | Tumour suppressor gene. Protein involved in post-replicative DNA mismatch repair system. | Sebaceous adenoma, cutaneous melanoma, uveal melanoma |
| MYD88 | 3p22.2 | Proto-oncogene. Adapter protein involved in *Toll-like* receptor and *IL-1* receptor signalling pathway in innate immune response. | Vitreoretinal lymphoma |

| | | | |
|---|---|---|---|
| NDP | Xp11.3 | Activates the canonical Wnt signalling pathway through *FZD4* and *LRP5* coreceptor. Involved in retinal vascularisation. | Coats, familial exudative vitreoretinopathy, persistent foetal vasculature, Norrie disease |
| NF1 | 17q11.2 | Tumour suppressor gene. Appears to be a negative regulator of the ras signal transduction pathway. | Multiple including, Lisch nodules, optic gliomas, capillary haemangiomas |
| NF2 | 22q12.2 | Tumour suppressor gene. Involved in regulation of contact-dependent inhibition of cell proliferation and functions in cell-cell adhesion and transmembrane signalling. | Hamartomas |
| NRAS | 1p13.2 | Proto-oncogene. Regulatory protein in cell division. | Conjunctival melanoma, uveal melanoma, cutaneous melanoma |
| PDCD1 | 2q37.3 | Tumour suppressor gene. Immune-inhibitory receptor expressed in activated T cells; involved in regulation T cell function. | Conjunctival squamous cell carcinoma, uveal melanoma |
| PLAG1 | 8q12.1 | Proto-oncogene. Involved in cell proliferation by regulating a wide array of target genes. | Pleomorphic adenoma lacrimal gland |
| PLCB4 | 20p12.3-p12.2 | Proto-oncogene. Encoding form of *phospholipase C* involved in *phosphoinositide cycle*. | Uveal melanoma |
| POT1 | 7q31.33 | Encodes a protein involved in telomere maintenance. | Uveal melanoma |
| PRDX3 | 10q26.11 | Mitochondrial protein with antioxidant function. | Uveal melanoma |
| PTCH1 | 9q22.32 | Tumour suppressor gene. Acts as a receptor for Sonic Hedgehog (*SHH*). | Ocular surface squamous cell neoplasia, retinoblastoma, basal cell |

| | | | |
|---|---|---|---|
| | | | carcinoma, Gorlin-Goltz syndrome, orbital teratoma |
| PTCH2 | 1q34.1 | Tumour suppressor gene. Role in control of cellular growth may be a receptor for *SHH*. | Gorlin-Goltz syndrome, |
| PTEN | 10q23.31 | Tumour suppressor gene. Acts as a dual-specificity protein phosphatase, dephosphorylating tyrosine-, serine- and threonine-phosphorylated proteins. | Cowden syndrome, retinoblastoma, conjunctival melanoma, uveal melanoma, PTEN hamartoma tumour syndrome, choroidal schwannoma |
| RB1 | 13q14.2 | Tumour suppressor gene. Key regulator of G1/S transition of cell cycle. | Retinoblastoma, ocular sebaceous carcinoma |
| RBL1 | 20q11.23 | Likely to be a tumour suppressor gene. Key regulator of entry into cell division. | Retinoblastoma |
| SF3B1 | 2q33.1 | Proto-oncogene. Involved in pre-mRNA splicing as a component of the splicing factor *SF3B* complex. | Uveal melanoma, conjunctival melanoma |
| SRSF2 | 17q25.1 | Proto-oncogene. Necessary for the regulation of gene transcription, pre-mRNA splicing, mRNA transport and stability. | Uveal melanoma, iris and ciliary melanocytoma |
| SUFU | 10q24.32 | Tumour suppressor gene. Negative regulator in the hedgehog/smoothened signalling pathway. | Retinoblastoma, conjunctival melanoma |
| TERT | 5p15.33 | Tumour suppressor gene. Encodes telomerase a ribonucleoprotein enzyme essential for the replication of chromosome termini in most eukaryotes. | Uveal melanoma, conjunctival melanoma |

| | | | |
|---|---|---|---|
| TP53 | 17p13.1 | Tumour suppressor gene implicated in many tumour types; induces growth arrest or apoptosis depending on physiological circumstances and cell type. | Ocular surface squamous cell neoplasia, Li-Fraumeni syndrome, orbital rhabdomyosarcoma, uveal melanoma |
| TSC1 | 9q34.13 | Tumour suppressor gene. Encodes growth inhibitory protein hamartin. | Tuberous sclerosis |
| TSC2 | 16p13.3 | Tumour suppressor gene. Encodes growth inhibitory protein tuberin. | Tuberous sclerosis |
| U2AF1 | 21q22.3 | Encodes an RNA-binding protein involved in the recognition of the 3’ splice site required for recruitment of U2 snRNP during pre-mRNA splicing. | Uveal melanoma |
| VHL | 3p25.3 | Tumour suppressor gene. Involved in ubiquitination and subsequent proteasomal degradation via von Hippel-Lindau ubiquitination complex. | Von Hippel-Lindau, Hemangioblastoma |
| XPA | 9q22.33 | Involved in DNA excision repair. | Ocular squamous cell carcinoma, pterygium, pinguecula, conjunctival melanoma |
