# Supplementary material for: Genetic analysis of ocular tumour-associated genes using large genomic datasets: insights into selection constraints and variant representation in the population
Source: BMJ Open Ophthalmol. 2024 Feb 21;9(1):e001565. doi: 10.1136/bmjophth-2023-001565 (PMC10897839; doi:10.1136/bmjophth-2023-001565)
Supplement: Supplementary data [file bmjophth-2023-001565supp002.pdf]

Supplementary Table 2: PANTHER Overrepresentation Test (Haploinsufficient Ocular Tumour-Associated Genes)

Analysis Type:				PANTHER Overrepresentation Test (Released 20221013)			
Annotation Version and Release Date:				GO Ontology database DOI: 10.5281/zenodo.7942786 Released 2023-05-10			
Analyzed List:				Client Text Box Input (Homo sapiens)			
Reference List:				Homo sapiens (all genes in database)			
Test Type:				FISHER			
Correction:				FDR			
GO biological process complete	Homo sapiens - REFLIST (20589)	Client Text Box Input (23)	Client Text Box Input (expected)	Client Text Box Input (over/under)	Client Text Box Input (fold Enrichment)	Client Text Box Input (raw P-value)	Client Text Box Input (FDR)
Schwann cell proliferation (GO:0014010)	3	2	.00	+	> 100	1.19E-05	1.14E-03
negative regulation of Schwann cell proliferation (GO:0010626)	8	3	.01	+	> 100	1.99E-07	5.24E-05
regulation of Schwann cell proliferation (GO:0010624)	9	3	.01	+	> 100	2.65E-07	6.34E-05
negative regulation of monocyte differentiation (GO:0045656)	7	2	.01	+	> 100	4.27E-05	3.08E-03
negative regulation of glial cell proliferation (GO:0060253)	16	4	.02	+	> 100	5.64E-09	2.92E-06
siRNA processing (GO:0030422)	8	2	.01	+	> 100	5.33E-05	3.70E-03
neural plate development (GO:0001840)	10	2	.01	+	> 100	7.81E-05	4.99E-03
negative regulation of oligodendrocyte differentiation (GO:0048715)	14	2	.02	+	> 100	1.42E-04	8.14E-03

regulation of glial cell proliferation (GO:0060251)	35	5	.04	+	> 100	6.97E-10	7.73E-07
negative regulation of protein import into nucleus (GO:0042308)	15	2	.02	+	> 100	1.60E-04	8.80E-03
negative regulation of cell division (GO:0051782)	16	2	.02	+	> 100	1.80E-04	9.72E-03
myeloid cell apoptotic process (GO:0033028)	16	2	.02	+	> 100	1.80E-04	9.69E-03
negative regulation of gliogenesis (GO:0014014)	42	5	.05	+	> 100	1.62E-09	1.39E-06
dorsal/ventral neural tube patterning (GO:0021904)	17	2	.02	+	> 100	2.01E-04	1.07E-02
regulation of monocyte differentiation (GO:0045655)	19	2	.02	+	94.23	2.47E-04	1.26E-02
ectoderm development (GO:0007398)	19	2	.02	+	94.23	2.47E-04	1.26E-02
glial cell proliferation (GO:0014009)	30	3	.03	+	89.52	6.48E-06	6.89E-04
negative regulation of stem cell proliferation (GO:2000647)	22	2	.02	+	81.38	3.24E-04	1.59E-02
sympathetic nervous system development (GO:0048485)	22	2	.02	+	81.38	3.24E-04	1.59E-02
negative regulation of cellular senescence (GO:2000773)	23	2	.03	+	77.84	3.52E-04	1.70E-02
smooth muscle tissue development (GO:0048745)	23	2	.03	+	77.84	3.52E-04	1.69E-02

negative regulation of nucleocytoplasmic transport (GO:0046823)	24	2	.03	+	74.60	3.81E-04	1.80E-02
negative regulation of smoothened signaling pathway (GO:0045879)	36	3	.04	+	74.60	1.08E-05	1.05E-03
positive regulation of mesenchymal cell proliferation (GO:0002053)	25	2	.03	+	71.61	4.11E-04	1.92E-02
regulation of vascular associated smooth muscle cell migration (GO:1904752)	25	2	.03	+	71.61	4.11E-04	1.91E-02
negative regulation of glial cell differentiation (GO:0045686)	26	2	.03	+	68.86	4.42E-04	2.03E-02
positive regulation of transcription regulatory region DNA binding (GO:2000679)	26	2	.03	+	68.86	4.42E-04	2.03E-02
negative regulation of myeloid leukocyte differentiation (GO:0002762)	53	4	.06	+	67.56	4.47E-07	8.58E-05
myelination in peripheral nervous system (GO:0022011)	29	2	.03	+	61.74	5.43E-04	2.40E-02
peripheral nervous system axon ensheathment (GO:0032292)	29	2	.03	+	61.74	5.43E-04	2.40E-02
spinal cord motor neuron differentiation (GO:0021522)	30	2	.03	+	59.68	5.79E-04	2.53E-02
negative regulation of endothelial cell	30	2	.03	+	59.68	5.79E-04	2.52E-02

apoptotic process (GO:2000352)							
branching involved in ureteric bud morphogenesis (GO:0001658)	46	3	.05	+	58.38	2.16E-05	1.82E-03
prostate gland development (GO:0030850)	46	3	.05	+	58.38	2.16E-05	1.81E-03
negative regulation of osteoblast differentiation (GO:0045668)	48	3	.05	+	55.95	2.44E-05	1.94E-03
regulation of stem cell proliferation (GO:0072091)	80	5	.09	+	55.95	3.36E-08	1.27E-05
Schwann cell development (GO:0014044)	32	2	.04	+	55.95	6.54E-04	2.83E-02
negative regulation of macroautophagy (GO:0016242)	32	2	.04	+	55.95	6.54E-04	2.82E-02
regulation of mesenchymal cell proliferation (GO:0010464)	32	2	.04	+	55.95	6.54E-04	2.81E-02
negative regulation of osteoclast differentiation (GO:0045671)	33	2	.04	+	54.25	6.93E-04	2.97E-02
neural tube patterning (GO:0021532)	33	2	.04	+	54.25	6.93E-04	2.96E-02
positive regulation of telomerase activity (GO:0051973)	34	2	.04	+	52.66	7.33E-04	3.11E-02
regulation of intrinsic apoptotic signaling pathway by p53 class mediator (GO:1902253)	34	2	.04	+	52.66	7.33E-04	3.10E-02
negative regulation of fibroblast	34	2	.04	+	52.66	7.33E-04	3.09E-02

proliferation (GO:0048147)							
regulation of gliogenesis (GO:0014013)	102	6	.11	+	52.66	1.69E-09	1.38E-06
hindlimb morphogenesis (GO:0035137)	35	2	.04	+	51.15	7.75E-04	3.26E-02
positive regulation of stress fiber assembly (GO:0051496)	53	3	.06	+	50.67	3.24E-05	2.45E-03
negative regulation of cell-matrix adhesion (GO:0001953)	36	2	.04	+	49.73	8.17E-04	3.41E-02
astrocyte development (GO:0014002)	36	2	.04	+	49.73	8.17E-04	3.40E-02
osteoclast differentiation (GO:0030316)	55	3	.06	+	48.83	3.60E-05	2.70E-03
Schwann cell differentiation (GO:0014037)	37	2	.04	+	48.39	8.61E-04	3.53E-02
positive regulation of vascular associated smooth muscle cell proliferation (GO:1904707)	37	2	.04	+	48.39	8.61E-04	3.52E-02
ureteric bud morphogenesis (GO:0060675)	57	3	.06	+	47.11	3.98E-05	2.95E-03
mesonephric tubule morphogenesis (GO:0072171)	58	3	.06	+	46.30	4.19E-05	3.05E-03
negative regulation of protein localization to nucleus (GO:1900181)	39	2	.04	+	45.91	9.51E-04	3.83E-02
negative regulation of epithelial cell apoptotic process (GO:1904036)	59	3	.07	+	45.52	4.40E-05	3.15E-03
endocardial cushion	40	2	.04	+	44.76	9.98E-04	3.96E-02

morphogenesis (GO:0003203)							
regulation of myeloid leukocyte differentiation (GO:0002761)	125	6	.14	+	42.97	5.43E-09	2.91E-06
negative regulation of myeloid cell differentiation (GO:0045638)	84	4	.09	+	42.63	2.58E-06	3.46E-04
positive regulation of G1/S transition of mitotic cell cycle (GO:1900087)	42	2	.05	+	42.63	1.10E-03	4.28E-02
T cell differentiation in thymus (GO:0033077)	63	3	.07	+	42.63	5.30E-05	3.71E-03
positive regulation of actin filament bundle assembly (GO:0032233)	63	3	.07	+	42.63	5.30E-05	3.69E-03
urogenital system development (GO:0001655)	64	3	.07	+	41.96	5.55E-05	3.83E-03
embryonic axis specification (GO:0000578)	43	2	.05	+	41.64	1.15E-03	4.45E-02
positive regulation of glucose transmembrane transport (GO:0010828)	43	2	.05	+	41.64	1.15E-03	4.44E-02
cell fate determination (GO:0001709)	44	2	.05	+	40.69	1.20E-03	4.59E-02
cell fate specification (GO:0001708)	88	4	.10	+	40.69	3.08E-06	3.99E-04
negative regulation of intracellular protein transport (GO:0090317)	44	2	.05	+	40.69	1.20E-03	4.58E-02
regulation of oligodendrocyte differentiation (GO:0048713)	44	2	.05	+	40.69	1.20E-03	4.57E-02

bone remodeling (GO:0046849)	44	2	.05	+	40.69	1.20E-03	4.55E-02
regulation of fibroblast proliferation (GO:0048145)	89	4	.10	+	40.23	3.22E-06	4.10E-04
regulation of cellular senescence (GO:2000772)	45	2	.05	+	39.79	1.25E-03	4.74E-02
autonomic nervous system development (GO:0048483)	45	2	.05	+	39.79	1.25E-03	4.73E-02
nephron tubule morphogenesis (GO:0072078)	68	3	.08	+	39.49	6.60E-05	4.36E-03
regulation of extrinsic apoptotic signaling pathway in absence of ligand (GO:2001239)	46	2	.05	+	38.92	1.30E-03	4.91E-02
ventral spinal cord development (GO:0021517)	46	2	.05	+	38.92	1.30E-03	4.90E-02
positive regulation of stem cell proliferation (GO:2000648)	46	2	.05	+	38.92	1.30E-03	4.88E-02
neural tube closure (GO:0001843)	93	4	.10	+	38.50	3.81E-06	4.59E-04
nephron epithelium morphogenesis (GO:0072088)	70	3	.08	+	38.36	7.17E-05	4.70E-03
tube closure (GO:0060606)	94	4	.11	+	38.09	3.97E-06	4.71E-04
regulation of osteoclast differentiation (GO:0045670)	72	3	.08	+	37.30	7.78E-05	5.05E-03
stem cell proliferation (GO:0072089)	72	3	.08	+	37.30	7.78E-05	5.03E-03
renal tubule morphogenesis (GO:0061333)	72	3	.08	+	37.30	7.78E-05	5.01E-03

heart valve development (GO:0003170)	72	3	.08	+	37.30	7.78E-05	4.99E-03
limbic system development (GO:0021761)	121	5	.14	+	36.99	2.43E-07	5.89E-05
nephron morphogenesis (GO:0072028)	73	3	.08	+	36.79	8.09E-05	5.11E-03
negative regulation of G1/S transition of mitotic cell cycle (GO:2000134)	74	3	.08	+	36.29	8.41E-05	5.29E-03
primary neural tube formation (GO:0014020)	99	4	.11	+	36.17	4.84E-06	5.37E-04
regulation of glial cell differentiation (GO:0045685)	75	3	.08	+	35.81	8.74E-05	5.47E-03
embryonic epithelial tube formation (GO:0001838)	127	5	.14	+	35.24	3.06E-07	6.89E-05
neural tube formation (GO:0001841)	106	4	.12	+	33.78	6.29E-06	6.73E-04
epithelial tube formation (GO:0072175)	135	5	.15	+	33.15	4.10E-07	8.28E-05
protein localization to chromosome (GO:0034502)	81	3	.09	+	33.15	1.09E-04	6.67E-03
negative regulation of leukocyte differentiation (GO:1902106)	109	4	.12	+	32.85	7.00E-06	7.29E-04
negative regulation of cell cycle G1/S phase transition (GO:1902807)	82	3	.09	+	32.75	1.13E-04	6.88E-03
dorsal/ventral pattern formation (GO:0009953)	84	3	.09	+	31.97	1.21E-04	7.26E-03
G1/S transition of mitotic cell cycle (GO:0000082)	84	3	.09	+	31.97	1.21E-04	7.23E-03

negative regulation of hemopoiesis (GO:1903707)	113	4	.13	+	31.69	8.03E-06	8.10E-04
nephron tubule development (GO:0072080)	86	3	.10	+	31.23	1.29E-04	7.64E-03
cell cycle G1/S phase transition (GO:0044843)	86	3	.10	+	31.23	1.29E-04	7.62E-03
ureteric bud development (GO:0001657)	88	3	.10	+	30.52	1.38E-04	8.04E-03
metanephros development (GO:0001656)	88	3	.10	+	30.52	1.38E-04	8.01E-03
associative learning (GO:0008306)	88	3	.10	+	30.52	1.38E-04	7.98E-03
negative regulation of neurogenesis (GO:0050768)	148	5	.17	+	30.24	6.38E-07	1.09E-04
mesonephric tubule development (GO:0072164)	89	3	.10	+	30.17	1.43E-04	8.16E-03
mesonephric epithelium development (GO:0072163)	89	3	.10	+	30.17	1.43E-04	8.13E-03
regulation of smoothened signaling pathway (GO:0008589)	89	3	.10	+	30.17	1.43E-04	8.10E-03
morphogenesis of embryonic epithelium (GO:0016331)	150	5	.17	+	29.84	6.80E-07	1.14E-04
tube formation (GO:0035148)	150	5	.17	+	29.84	6.80E-07	1.12E-04
positive regulation of mitotic cell cycle phase transition (GO:1901992)	90	3	.10	+	29.84	1.47E-04	8.30E-03
kidney morphogenesis (GO:0060993)	90	3	.10	+	29.84	1.47E-04	8.27E-03

smoothened signaling pathway (GO:0007224)	91	3	.10	+	29.51	1.52E-04	8.47E-03
renal tubule development (GO:0061326)	91	3	.10	+	29.51	1.52E-04	8.44E-03
hippocampus development (GO:0021766)	92	3	.10	+	29.19	1.57E-04	8.68E-03
regulation of stress fiber assembly (GO:0051492)	92	3	.10	+	29.19	1.57E-04	8.65E-03
regulation of cell-matrix adhesion (GO:0001952)	123	4	.14	+	29.11	1.11E-05	1.07E-03
mesonephros development (GO:0001823)	93	3	.10	+	28.88	1.62E-04	8.86E-03
negative regulation of nervous system development (GO:0051961)	155	5	.17	+	28.88	7.96E-07	1.26E-04
regulation of osteoblast differentiation (GO:0045667)	127	4	.14	+	28.19	1.26E-05	1.20E-03
neural tube development (GO:0021915)	163	5	.18	+	27.46	1.01E-06	1.59E-04
regulation of epithelial cell apoptotic process (GO:1904035)	99	3	.11	+	27.13	1.94E-04	1.03E-02
negative regulation of protein kinase activity (GO:0006469)	200	6	.22	+	26.86	8.12E-08	2.57E-05
cellular response to decreased oxygen levels (GO:0036294)	134	4	.15	+	26.72	1.54E-05	1.42E-03
regulation of myeloid cell differentiation (GO:0045637)	202	6	.23	+	26.59	8.59E-08	2.62E-05
regulation of actomyosin structure	101	3	.11	+	26.59	2.05E-04	1.08E-02

organization (GO:0110020)							
regulation of neural precursor cell proliferation (GO:2000177)	101	3	.11	+	26.59	2.05E-04	1.08E-02
spinal cord development (GO:0021510)	102	3	.11	+	26.33	2.11E-04	1.11E-02
tissue remodeling (GO:0048771)	104	3	.12	+	25.82	2.23E-04	1.16E-02
negative regulation of epithelial cell proliferation (GO:0050680)	140	4	.16	+	25.58	1.82E-05	1.62E-03
regulation of actin filament bundle assembly (GO:0032231)	105	3	.12	+	25.58	2.30E-04	1.18E-02
nephron epithelium development (GO:0072009)	109	3	.12	+	24.64	2.56E-04	1.29E-02
negative regulation of protein serine/threonine kinase activity (GO:0071901)	110	3	.12	+	24.41	2.62E-04	1.32E-02
negative regulation of kinase activity (GO:0033673)	222	6	.25	+	24.19	1.48E-07	4.33E-05
regulation of protein localization to nucleus (GO:1900180)	148	4	.17	+	24.19	2.26E-05	1.86E-03
myeloid leukocyte differentiation (GO:0002573)	150	4	.17	+	23.87	2.38E-05	1.92E-03
cellular response to oxygen levels (GO:0071453)	150	4	.17	+	23.87	2.38E-05	1.91E-03
positive regulation of cell cycle phase transition (GO:1901989)	113	3	.13	+	23.77	2.83E-04	1.42E-02

glial cell development (GO:0021782)	114	3	.13	+	23.56	2.91E-04	1.45E-02
myelination (GO:0042552)	115	3	.13	+	23.35	2.98E-04	1.48E-02
pallium development (GO:0021543)	193	5	.22	+	23.19	2.27E-06	3.10E-04
ensheathment of neurons (GO:0007272)	117	3	.13	+	22.95	3.13E-04	1.55E-02
axon ensheathment (GO:0008366)	117	3	.13	+	22.95	3.13E-04	1.54E-02
negative regulation of neuron apoptotic process (GO:0043524)	158	4	.18	+	22.66	2.90E-05	2.25E-03
negative regulation of cell development (GO:0010721)	279	7	.31	+	22.46	1.87E-08	7.62E-06
positive regulation of mitotic cell cycle (GO:0045931)	120	3	.13	+	22.38	3.37E-04	1.63E-02
regulation of G1/S transition of mitotic cell cycle (GO:2000045)	164	4	.18	+	21.83	3.34E-05	2.52E-03
cellular response to hypoxia (GO:0071456)	125	3	.14	+	21.48	3.78E-04	1.80E-02
mitotic cell cycle checkpoint signaling (GO:0007093)	127	3	.14	+	21.15	3.96E-04	1.85E-02
negative regulation of mitotic cell cycle (GO:0045930)	214	5	.24	+	20.92	3.72E-06	4.59E-04
cell fate commitment (GO:0045165)	258	6	.29	+	20.82	3.51E-07	7.56E-05
positive regulation of cell-substrate adhesion (GO:0010811)	129	3	.14	+	20.82	4.14E-04	1.92E-02
MAPK cascade (GO:0000165)	216	5	.24	+	20.72	3.89E-06	4.65E-04

cerebral cortex development (GO:0021987)	130	3	.15	+	20.66	4.23E-04	1.96E-02
epithelial cell proliferation (GO:0050673)	174	4	.19	+	20.58	4.19E-05	3.04E-03
endocrine system development (GO:0035270)	131	3	.15	+	20.50	4.33E-04	1.99E-02
positive regulation of supramolecular fiber organization (GO:1902905)	175	4	.20	+	20.46	4.28E-05	3.08E-03
regulation of cell-substrate adhesion (GO:0010810)	219	5	.24	+	20.44	4.16E-06	4.82E-04
negative regulation of transferase activity (GO:0051348)	266	6	.30	+	20.19	4.18E-07	8.32E-05
gliogenesis (GO:0042063)	266	6	.30	+	20.19	4.18E-07	8.21E-05
regulation of neuron apoptotic process (GO:0043523)	226	5	.25	+	19.80	4.83E-06	5.40E-04
T cell differentiation (GO:0030217)	181	4	.20	+	19.78	4.87E-05	3.42E-03
myeloid cell homeostasis (GO:0002262)	136	3	.15	+	19.75	4.81E-04	2.19E-02
branching morphogenesis of an epithelial tube (GO:0048754)	137	3	.15	+	19.60	4.92E-04	2.22E-02
regulation of leukocyte differentiation (GO:1902105)	321	7	.36	+	19.52	4.77E-08	1.58E-05
nephron development (GO:0072006)	138	3	.15	+	19.46	5.02E-04	2.26E-02
kidney epithelium development (GO:0072073)	140	3	.16	+	19.18	5.23E-04	2.33E-02
regulation of smooth muscle	142	3	.16	+	18.91	5.45E-04	2.40E-02

cell proliferation (GO:0048660)							
response to ionizing radiation (GO:0010212)	143	3	.16	+	18.78	5.56E-04	2.43E-02
regulation of cell cycle G1/S phase transition (GO:1902806)	191	4	.21	+	18.75	5.97E-05	4.02E-03
regulation of neuron death (GO:1901214)	292	6	.33	+	18.39	7.13E-07	1.15E-04
negative regulation of neuron death (GO:1901215)	195	4	.22	+	18.36	6.46E-05	4.31E-03
glial cell differentiation (GO:0010001)	206	4	.23	+	17.38	7.96E-05	5.07E-03
learning (GO:0007612)	156	3	.17	+	17.21	7.12E-04	3.03E-02
epithelial tube morphogenesis (GO:0060562)	316	6	.35	+	17.00	1.12E-06	1.72E-04
leukocyte differentiation (GO:0002521)	424	8	.47	+	16.89	1.30E-08	5.45E-06
negative regulation of protein phosphorylation (GO:0001933)	318	6	.36	+	16.89	1.16E-06	1.75E-04
cell cycle checkpoint signaling (GO:0000075)	161	3	.18	+	16.68	7.79E-04	3.26E-02
immune system development (GO:0002520)	165	3	.18	+	16.28	8.35E-04	3.46E-02
mitotic cell cycle phase transition (GO:0044772)	165	3	.18	+	16.28	8.35E-04	3.45E-02
morphogenesis of a branching epithelium (GO:0061138)	165	3	.18	+	16.28	8.35E-04	3.44E-02
regulation of neurogenesis (GO:0050767)	386	7	.43	+	16.23	1.64E-07	4.54E-05
negative regulation of	167	3	.19	+	16.08	8.65E-04	3.52E-02

mitotic cell cycle phase transition (GO:1901991)							
anatomical structure maturation (GO:0071695)	223	4	.25	+	16.06	1.08E-04	6.61E-03
telencephalon development (GO:0021537)	279	5	.31	+	16.04	1.32E-05	1.25E-03
negative regulation of MAPK cascade (GO:0043409)	171	3	.19	+	15.70	9.25E-04	3.74E-02
negative regulation of cell cycle phase transition (GO:1901988)	228	4	.25	+	15.70	1.17E-04	7.07E-03
response to decreased oxygen levels (GO:0036293)	286	5	.32	+	15.65	1.48E-05	1.38E-03
myeloid cell differentiation (GO:0030099)	286	5	.32	+	15.65	1.48E-05	1.37E-03
negative regulation of apoptotic signaling pathway (GO:2001234)	229	4	.26	+	15.64	1.19E-04	7.16E-03
negative regulation of Wnt signaling pathway (GO:0030178)	172	3	.19	+	15.61	9.40E-04	3.79E-02
regulation of hemopoiesis (GO:1903706)	403	7	.45	+	15.55	2.18E-07	5.55E-05
negative regulation of phosphorylation (GO:0042326)	348	6	.39	+	15.43	1.94E-06	2.71E-04
lymphocyte differentiation (GO:0030098)	290	5	.32	+	15.43	1.58E-05	1.44E-03
morphogenesis of a branching structure (GO:0001763)	175	3	.20	+	15.35	9.88E-04	3.94E-02
cell maturation (GO:0048469)	175	3	.20	+	15.35	9.88E-04	3.93E-02

cell cycle phase transition (GO:0044770)	176	3	.20	+	15.26	1.00E-03	3.98E-02
negative regulation of DNA-binding transcription factor activity (GO:0043433)	176	3	.20	+	15.26	1.00E-03	3.97E-02
negative regulation of cell population proliferation (GO:0008285)	708	12	.79	+	15.17	2.86E-12	7.41E-09
positive regulation of binding (GO:0051099)	179	3	.20	+	15.00	1.05E-03	4.14E-02
positive regulation of neurogenesis (GO:0050769)	240	4	.27	+	14.92	1.42E-04	8.14E-03
negative regulation of cell cycle (GO:0045786)	361	6	.40	+	14.88	2.39E-06	3.23E-04
kidney development (GO:0001822)	303	5	.34	+	14.77	1.95E-05	1.69E-03
cell-cell junction organization (GO:0045216)	182	3	.20	+	14.76	1.10E-03	4.30E-02
cell-substrate adhesion (GO:0031589)	185	3	.21	+	14.52	1.16E-03	4.47E-02
regulation of mitotic cell cycle (GO:0007346)	494	8	.55	+	14.50	4.16E-08	1.47E-05
positive regulation of cytoskeleton organization (GO:0051495)	186	3	.21	+	14.44	1.17E-03	4.52E-02
regulation of epithelial cell proliferation (GO:0050678)	372	6	.42	+	14.44	2.83E-06	3.76E-04
regulation of apoptotic signaling pathway (GO:2001233)	375	6	.42	+	14.32	2.97E-06	3.90E-04
regulation of chromosome	250	4	.28	+	14.32	1.66E-04	9.00E-03

organization (GO:0033044)							
heart development (GO:0007507)	563	9	.63	+	14.31	5.30E-09	2.94E-06
renal system development (GO:0072001)	313	5	.35	+	14.30	2.27E-05	1.86E-03
response to oxygen levels (GO:0070482)	314	5	.35	+	14.25	2.30E-05	1.88E-03
response to light stimulus (GO:0009416)	316	5	.35	+	14.16	2.37E-05	1.93E-03
tissue morphogenesis (GO:0048729)	570	9	.64	+	14.13	5.89E-09	2.95E-06
cellular response to xenobiotic stimulus (GO:0071466)	191	3	.21	+	14.06	1.27E-03	4.78E-02
heart morphogenesis (GO:0003007)	255	4	.28	+	14.04	1.79E-04	9.67E-03
protein localization to nucleus (GO:0034504)	193	3	.22	+	13.91	1.30E-03	4.88E-02
negative regulation of protein modification process (GO:0031400)	462	7	.52	+	13.56	5.40E-07	9.98E-05
regulation of nervous system development (GO:0051960)	466	7	.52	+	13.45	5.72E-07	1.02E-04
regulation of mitotic cell cycle phase transition (GO:1901990)	333	5	.37	+	13.44	3.04E-05	2.35E-03
regulation of Wnt signaling pathway (GO:0030111)	334	5	.37	+	13.40	3.08E-05	2.37E-03
mononuclear cell differentiation (GO:1903131)	335	5	.37	+	13.36	3.12E-05	2.39E-03
regulation of protein stability (GO:0031647)	335	5	.37	+	13.36	3.12E-05	2.38E-03

negative regulation of phosphate metabolic process (GO:0045936)	404	6	.45	+	13.29	4.53E-06	5.13E-04
negative regulation of phosphorus metabolic process (GO:0010563)	405	6	.45	+	13.26	4.59E-06	5.16E-04
response to hypoxia (GO:0001666)	272	4	.30	+	13.16	2.28E-04	1.18E-02
negative regulation of cell cycle process (GO:0010948)	274	4	.31	+	13.07	2.34E-04	1.20E-02
developmental maturation (GO:0021700)	278	4	.31	+	12.88	2.47E-04	1.26E-02
negative regulation of cell migration (GO:0030336)	286	4	.32	+	12.52	2.75E-04	1.38E-02
positive regulation of nervous system development (GO:0051962)	289	4	.32	+	12.39	2.86E-04	1.43E-02
regulation of cell cycle phase transition (GO:1901987)	434	6	.48	+	12.38	6.78E-06	7.12E-04
response to radiation (GO:0009314)	445	6	.50	+	12.07	7.81E-06	7.98E-04
negative regulation of cell differentiation (GO:0045596)	670	9	.75	+	12.02	2.34E-08	9.32E-06
mitotic cell cycle process (GO:1903047)	522	7	.58	+	12.00	1.21E-06	1.79E-04
negative regulation of cell motility (GO:2000146)	301	4	.34	+	11.90	3.33E-04	1.63E-02
reproductive structure development (GO:0048608)	301	4	.34	+	11.90	3.33E-04	1.62E-02

hemopoiesis (GO:0030097)	685	9	.77	+	11.76	2.83E-08	1.10E-05
regulation of supramolecular fiber organization (GO:1902903)	381	5	.43	+	11.75	5.72E-05	3.93E-03
reproductive system development (GO:0061458)	305	4	.34	+	11.74	3.50E-04	1.69E-02
morphogenesis of an epithelium (GO:0002009)	458	6	.51	+	11.73	9.19E-06	9.09E-04
mitotic cell cycle (GO:0000278)	616	8	.69	+	11.63	2.21E-07	5.45E-05
T cell activation (GO:0042110)	315	4	.35	+	11.37	3.95E-04	1.85E-02
regulation of cell cycle (GO:0051726)	1120	14	1.25	+	11.19	1.10E-12	3.43E-09
regulation of cell cycle process (GO:0010564)	725	9	.81	+	11.11	4.58E-08	1.55E-05
forebrain development (GO:0030900)	409	5	.46	+	10.94	7.96E-05	5.05E-03
chordate embryonic development (GO:0043009)	661	8	.74	+	10.83	3.76E-07	7.68E-05
regulation of cell development (GO:0060284)	829	10	.93	+	10.80	8.36E-09	3.61E-06
muscle tissue development (GO:0060537)	334	4	.37	+	10.72	4.92E-04	2.23E-02
camera-type eye development (GO:0043010)	337	4	.38	+	10.63	5.08E-04	2.28E-02
negative regulation of locomotion (GO:0040013)	338	4	.38	+	10.59	5.14E-04	2.30E-02
gland development (GO:0048732)	425	5	.47	+	10.53	9.52E-05	5.92E-03
regulation of intracellular transport (GO:0032386)	340	4	.38	+	10.53	5.25E-04	2.33E-02

embryo development ending in birth or egg hatching (GO:0009792)	683	8	.76	+	10.49	4.81E-07	9.10E-05
response to xenobiotic stimulus (GO:0009410)	433	5	.48	+	10.34	1.04E-04	6.40E-03
negative regulation of intracellular signal transduction (GO:1902532)	524	6	.59	+	10.25	1.95E-05	1.69E-03
regulation of protein kinase activity (GO:0045859)	615	7	.69	+	10.19	3.54E-06	4.44E-04
positive regulation of cell development (GO:0010720)	441	5	.49	+	10.15	1.13E-04	6.86E-03
negative regulation of catalytic activity (GO:0043086)	630	7	.70	+	9.95	4.15E-06	4.84E-04
chromosome organization (GO:0051276)	456	5	.51	+	9.82	1.32E-04	7.74E-03
regulation of transferase activity (GO:0051338)	847	9	.95	+	9.51	1.70E-07	4.64E-05
apoptotic process (GO:0006915)	1047	11	1.17	+	9.40	4.74E-09	2.94E-06
positive regulation of cell differentiation (GO:0045597)	857	9	.96	+	9.40	1.88E-07	5.03E-05
lymphocyte activation (GO:0046649)	478	5	.53	+	9.36	1.64E-04	8.95E-03
eye development (GO:0001654)	385	4	.43	+	9.30	8.32E-04	3.46E-02
visual system development (GO:0150063)	389	4	.43	+	9.20	8.65E-04	3.52E-02
tube morphogenesis (GO:0035239)	684	7	.76	+	9.16	7.08E-06	7.28E-04

regulation of cell population proliferation (GO:0042127)	1674	17	1.87	+	9.09	2.01E-14	3.12E-10
embryonic morphogenesis (GO:0048598)	591	6	.66	+	9.09	3.82E-05	2.85E-03
sensory system development (GO:0048880)	395	4	.44	+	9.07	9.15E-04	3.71E-02
animal organ morphogenesis (GO:0009887)	991	10	1.11	+	9.03	4.48E-08	1.55E-05
programmed cell death (GO:0012501)	1093	11	1.22	+	9.01	7.39E-09	3.48E-06
in utero embryonic development (GO:0001701)	401	4	.45	+	8.93	9.67E-04	3.88E-02
cell death (GO:0008219)	1105	11	1.23	+	8.91	8.27E-09	3.67E-06
regulation of kinase activity (GO:0043549)	709	7	.79	+	8.84	8.93E-06	8.89E-04
positive regulation of organelle organization (GO:0010638)	508	5	.57	+	8.81	2.17E-04	1.13E-02
negative regulation of developmental process (GO:0051093)	919	9	1.03	+	8.77	3.38E-07	7.39E-05
circulatory system development (GO:0072359)	926	9	1.03	+	8.70	3.60E-07	7.66E-05
regulation of cell differentiation (GO:0045595)	1558	15	1.74	+	8.62	4.44E-12	9.85E-09
cell population proliferation (GO:0008283)	729	7	.81	+	8.60	1.07E-05	1.04E-03
negative regulation of signal transduction (GO:0009968)	1266	12	1.41	+	8.49	2.19E-09	1.62E-06
negative regulation of molecular	975	9	1.09	+	8.26	5.54E-07	1.00E-04

function (GO:0044092)							
regulation of protein localization (GO:0032880)	874	8	.98	+	8.19	3.01E-06	3.93E-04
negative regulation of transcription by RNA polymerase II (GO:0000122)	985	9	1.10	+	8.18	6.03E-07	1.06E-04
negative regulation of protein metabolic process (GO:0051248)	986	9	1.10	+	8.17	6.08E-07	1.06E-04
cell cycle process (GO:0022402)	881	8	.98	+	8.13	3.19E-06	4.10E-04
negative regulation of apoptotic process (GO:0043066)	892	8	1.00	+	8.03	3.50E-06	4.42E-04
response to abiotic stimulus (GO:0009628)	1118	10	1.25	+	8.01	1.38E-07	4.11E-05
tube development (GO:0035295)	900	8	1.01	+	7.96	3.73E-06	4.57E-04
negative regulation of cell communication (GO:0010648)	1361	12	1.52	+	7.89	4.94E-09	2.95E-06
negative regulation of signaling (GO:0023057)	1362	12	1.52	+	7.89	4.98E-09	2.87E-06
positive regulation of hydrolase activity (GO:0051345)	571	5	.64	+	7.84	3.71E-04	1.77E-02
cell morphogenesis (GO:0000902)	687	6	.77	+	7.82	8.74E-05	5.45E-03
negative regulation of programmed cell death (GO:0043069)	920	8	1.03	+	7.78	4.39E-06	5.05E-04
regulation of multicellular organismal	1401	12	1.57	+	7.67	6.84E-09	3.32E-06

development (GO:2000026)							
embryo development (GO:0009790)	1054	9	1.18	+	7.64	1.06E-06	1.64E-04
positive regulation of developmental process (GO:0051094)	1319	11	1.47	+	7.47	5.09E-08	1.65E-05
anatomical structure formation involved in morphogenesis (GO:0048646)	961	8	1.07	+	7.45	6.04E-06	6.52E-04
leukocyte activation (GO:0045321)	601	5	.67	+	7.45	4.68E-04	2.13E-02
head development (GO:0060322)	847	7	.95	+	7.40	2.80E-05	2.18E-03
protein localization to organelle (GO:0033365)	731	6	.82	+	7.35	1.23E-04	7.27E-03
epithelium development (GO:0060429)	1103	9	1.23	+	7.30	1.54E-06	2.19E-04
regulation of cellular localization (GO:0060341)	981	8	1.10	+	7.30	7.03E-06	7.27E-04
negative regulation of cellular metabolic process (GO:0031324)	2217	18	2.48	+	7.27	7.94E-14	6.16E-10
negative regulation of DNA-templated transcription (GO:0045892)	1327	10	1.48	+	6.75	6.69E-07	1.13E-04
brain development (GO:0007420)	798	6	.89	+	6.73	1.97E-04	1.05E-02
regulation of apoptotic process (GO:0042981)	1471	11	1.64	+	6.69	1.54E-07	4.35E-05
regulation of protein	1071	8	1.20	+	6.69	1.33E-05	1.25E-03

phosphorylation (GO:0001932)							
negative regulation of RNA biosynthetic process (GO:1902679)	1341	10	1.50	+	6.68	7.36E-07	1.18E-04
regulation of MAPK cascade (GO:0043408)	672	5	.75	+	6.66	7.75E-04	3.25E-02
positive regulation of cell population proliferation (GO:0008284)	950	7	1.06	+	6.60	5.80E-05	3.95E-03
negative regulation of response to stimulus (GO:0048585)	1637	12	1.83	+	6.56	3.88E-08	1.40E-05
regulation of programmed cell death (GO:0043067)	1521	11	1.70	+	6.47	2.16E-07	5.60E-05
positive regulation of catalytic activity (GO:0043085)	1112	8	1.24	+	6.44	1.75E-05	1.59E-03
negative regulation of nitrogen compound metabolic process (GO:0051172)	2376	17	2.65	+	6.40	6.08E-12	1.18E-08
negative regulation of multicellular organismal process (GO:0051241)	1125	8	1.26	+	6.37	1.90E-05	1.67E-03
cell cycle (GO:0007049)	1270	9	1.42	+	6.34	4.90E-06	5.39E-04
regulation of cell motility (GO:2000145)	991	7	1.11	+	6.32	7.56E-05	4.93E-03
intracellular signal transduction (GO:0035556)	1558	11	1.74	+	6.32	2.75E-07	6.48E-05
negative regulation of macromolecule biosynthetic	1560	11	1.74	+	6.31	2.79E-07	6.47E-05

process (GO:0010558)							
negative regulation of nucleobase-containing compound metabolic process (GO:0045934)	1568	11	1.75	+	6.28	2.94E-07	6.71E-05
regulation of protein modification process (GO:0031399)	1443	10	1.61	+	6.20	1.44E-06	2.08E-04
cell activation (GO:0001775)	724	5	.81	+	6.18	1.08E-03	4.24E-02
negative regulation of macromolecule metabolic process (GO:0010605)	2755	19	3.08	+	6.17	1.38E-13	7.12E-10
negative regulation of RNA metabolic process (GO:0051253)	1451	10	1.62	+	6.17	1.51E-06	2.17E-04
negative regulation of cellular biosynthetic process (GO:0031327)	1601	11	1.79	+	6.15	3.62E-07	7.60E-05
tissue development (GO:0009888)	1753	12	1.96	+	6.13	8.28E-08	2.57E-05
regulation of phosphorylation (GO:0042325)	1170	8	1.31	+	6.12	2.52E-05	2.00E-03
regulation of organelle organization (GO:0033043)	1175	8	1.31	+	6.09	2.60E-05	2.04E-03
regulation of locomotion (GO:0040012)	1036	7	1.16	+	6.05	9.99E-05	6.18E-03
anatomical structure morphogenesis (GO:0009653)	2244	15	2.51	+	5.98	7.86E-10	8.14E-07
negative regulation of biosynthetic	1650	11	1.84	+	5.97	4.90E-07	9.17E-05

process (GO:0009890)							
central nervous system development (GO:0007417)	1069	7	1.19	+	5.86	1.21E-04	7.22E-03
regulation of developmental process (GO:0050793)	2462	16	2.75	+	5.82	1.94E-10	3.02E-07
regulation of cell migration (GO:0030334)	933	6	1.04	+	5.76	4.55E-04	2.08E-02
negative regulation of metabolic process (GO:0009892)	2985	19	3.33	+	5.70	5.98E-13	2.32E-09
negative regulation of gene expression (GO:0010629)	955	6	1.07	+	5.62	5.15E-04	2.30E-02
regulation of cellular component biogenesis (GO:0044087)	968	6	1.08	+	5.55	5.53E-04	2.43E-02
regulation of phosphate metabolic process (GO:0019220)	1315	8	1.47	+	5.45	5.79E-05	3.96E-03
generation of neurons (GO:0048699)	1151	7	1.29	+	5.44	1.92E-04	1.03E-02
regulation of phosphorus metabolic process (GO:0051174)	1316	8	1.47	+	5.44	5.82E-05	3.95E-03
neurogenesis (GO:0022008)	1321	8	1.48	+	5.42	5.98E-05	4.01E-03
regulation of catalytic activity (GO:0050790)	1827	11	2.04	+	5.39	1.35E-06	1.98E-04
positive regulation of gene expression (GO:0010628)	1173	7	1.31	+	5.34	2.16E-04	1.13E-02
positive regulation of molecular function (GO:0044093)	1519	9	1.70	+	5.30	2.08E-05	1.75E-03

regulation of multicellular organismal process (GO:0051239)	2983	17	3.33	+	5.10	2.37E-10	3.07E-07
cell-cell signaling (GO:0007267)	1092	6	1.22	+	4.92	1.04E-03	4.10E-02
animal organ development (GO:0048513)	2925	16	3.27	+	4.90	2.56E-09	1.73E-06
positive regulation of multicellular organismal process (GO:0051240)	1654	9	1.85	+	4.87	4.08E-05	2.99E-03
regulation of immune system process (GO:0002682)	1488	8	1.66	+	4.81	1.38E-04	8.06E-03
positive regulation of cellular component organization (GO:0051130)	1118	6	1.25	+	4.80	1.17E-03	4.51E-02
regulation of protein metabolic process (GO:0051246)	2462	13	2.75	+	4.73	3.71E-07	7.69E-05
cell surface receptor signaling pathway (GO:0007166)	2093	11	2.34	+	4.70	5.15E-06	5.63E-04
cellular response to oxygen-containing compound (GO:1901701)	1146	6	1.28	+	4.69	1.33E-03	4.97E-02
protein localization (GO:0008104)	1936	10	2.16	+	4.62	1.99E-05	1.70E-03
cellular macromolecule localization (GO:0070727)	1942	10	2.17	+	4.61	2.04E-05	1.73E-03
regulation of transport (GO:0051049)	1776	9	1.98	+	4.54	7.14E-05	4.70E-03
regulation of molecular function (GO:0065009)	2569	13	2.87	+	4.53	6.11E-07	1.05E-04

cell development (GO:0048468)	2185	11	2.44	+	4.51	7.82E-06	7.94E-04
nervous system development (GO:0007399)	2233	11	2.49	+	4.41	9.65E-06	9.48E-04
system development (GO:0048731)	3553	17	3.97	+	4.28	3.83E-09	2.48E-06
regulation of localization (GO:0032879)	2131	10	2.38	+	4.20	4.58E-05	3.25E-03
regulation of signal transduction (GO:0009966)	2992	14	3.34	+	4.19	4.24E-07	8.23E-05
positive regulation of DNA-templated transcription (GO:0045893)	1716	8	1.92	+	4.17	3.68E-04	1.76E-02
positive regulation of RNA biosynthetic process (GO:1902680)	1723	8	1.92	+	4.16	3.78E-04	1.79E-02
regulation of intracellular signal transduction (GO:1902531)	1727	8	1.93	+	4.15	3.84E-04	1.81E-02
macromolecule localization (GO:0033036)	2377	11	2.66	+	4.14	1.76E-05	1.58E-03
multicellular organism development (GO:0007275)	3966	18	4.43	+	4.06	1.69E-09	1.31E-06
positive regulation of nucleobase-containing compound metabolic process (GO:0045935)	2052	9	2.29	+	3.93	2.18E-04	1.13E-02
positive regulation of RNA metabolic process (GO:0051254)	1856	8	2.07	+	3.86	6.25E-04	2.71E-02
regulation of transcription by RNA polymerase II (GO:0006357)	2594	11	2.90	+	3.80	4.03E-05	2.97E-03

regulation of signaling (GO:0023051)	3396	14	3.79	+	3.69	2.05E-06	2.85E-04
positive regulation of macromolecule biosynthetic process (GO:0010557)	1942	8	2.17	+	3.69	8.45E-04	3.47E-02
regulation of cell communication (GO:0010646)	3405	14	3.80	+	3.68	2.12E-06	2.92E-04
regulation of cellular component organization (GO:0051128)	2439	10	2.72	+	3.67	1.45E-04	8.19E-03
cellular localization (GO:0051641)	2700	11	3.02	+	3.65	5.88E-05	3.97E-03
regulation of response to stimulus (GO:0048583)	3960	16	4.42	+	3.62	2.18E-07	5.46E-05
negative regulation of cellular process (GO:0048523)	4705	19	5.26	+	3.61	2.26E-09	1.60E-06
protein-containing complex organization (GO:0043933)	1984	8	2.22	+	3.61	9.74E-04	3.90E-02
positive regulation of cellular metabolic process (GO:0031325)	2976	12	3.32	+	3.61	2.39E-05	1.91E-03
positive regulation of cellular biosynthetic process (GO:0031328)	2022	8	2.26	+	3.54	1.10E-03	4.31E-02
positive regulation of metabolic process (GO:0009893)	3825	15	4.27	+	3.51	1.18E-06	1.76E-04
cell differentiation (GO:0030154)	3590	14	4.01	+	3.49	4.07E-06	4.78E-04
cellular developmental process (GO:0048869)	3614	14	4.04	+	3.47	4.41E-06	5.04E-04

positive regulation of nitrogen compound metabolic process (GO:0051173)	3121	12	3.49	+	3.44	3.89E-05	2.89E-03
positive regulation of biosynthetic process (GO:0009891)	2081	8	2.32	+	3.44	1.33E-03	4.98E-02
regulation of cellular metabolic process (GO:0031323)	5482	21	6.12	+	3.43	1.24E-10	2.13E-07
regulation of DNA-templated transcription (GO:0006355)	3450	13	3.85	+	3.37	1.77E-05	1.58E-03
negative regulation of biological process (GO:0048519)	5320	20	5.94	+	3.37	1.37E-09	1.33E-06
cellular response to chemical stimulus (GO:0070887)	2397	9	2.68	+	3.36	6.99E-04	2.98E-02
regulation of RNA biosynthetic process (GO:2001141)	3473	13	3.88	+	3.35	1.91E-05	1.67E-03
regulation of nitrogen compound metabolic process (GO:0051171)	5646	21	6.31	+	3.33	2.25E-10	3.17E-07
positive regulation of macromolecule metabolic process (GO:0010604)	3514	13	3.93	+	3.31	2.18E-05	1.81E-03
regulation of primary metabolic process (GO:0080090)	5825	21	6.51	+	3.23	4.23E-10	5.05E-07
regulation of macromolecule biosynthetic process (GO:0010556)	3961	14	4.42	+	3.16	1.34E-05	1.25E-03
regulation of gene expression (GO:0010468)	4888	17	5.46	+	3.11	5.45E-07	9.95E-05

anatomical structure development (GO:0048856)	5201	18	5.81	+	3.10	1.54E-07	4.43E-05
regulation of nucleobase-containing compound metabolic process (GO:0019219)	4051	14	4.53	+	3.09	1.76E-05	1.59E-03
regulation of RNA metabolic process (GO:0051252)	3766	13	4.21	+	3.09	4.67E-05	3.30E-03
positive regulation of cellular process (GO:0048522)	5511	19	6.16	+	3.09	3.73E-08	1.38E-05
regulation of cellular biosynthetic process (GO:0031326)	4068	14	4.54	+	3.08	1.85E-05	1.63E-03
regulation of macromolecule metabolic process (GO:0060255)	6188	21	6.91	+	3.04	1.43E-09	1.31E-06
regulation of biosynthetic process (GO:0009889)	4177	14	4.67	+	3.00	2.53E-05	2.00E-03
signal transduction (GO:0007165)	4819	16	5.38	+	2.97	3.56E-06	4.43E-04
cell communication (GO:0007154)	5278	17	5.90	+	2.88	1.74E-06	2.46E-04
developmental process (GO:0032502)	5702	18	6.37	+	2.83	6.89E-07	1.13E-04
signaling (GO:0023052)	5119	16	5.72	+	2.80	8.27E-06	8.28E-04
regulation of metabolic process (GO:0019222)	6719	21	7.51	+	2.80	7.50E-09	3.43E-06
response to chemical (GO:0042221)	3899	12	4.36	+	2.76	3.57E-04	1.71E-02
cellular component organization (GO:0016043)	5560	17	6.21	+	2.74	3.81E-06	4.62E-04

positive regulation of biological process (GO:0048518)	6219	19	6.95	+	2.73	3.09E-07	6.86E-05
cellular component organization or biogenesis (GO:0071840)	5774	17	6.45	+	2.64	6.68E-06	7.05E-04
multicellular organismal process (GO:0032501)	6713	19	7.50	+	2.53	1.16E-06	1.76E-04
cellular response to stimulus (GO:0051716)	6472	18	7.23	+	2.49	5.26E-06	5.71E-04
response to stimulus (GO:0050896)	8243	19	9.21	+	2.06	4.47E-05	3.18E-03
regulation of cellular process (GO:0050794)	11039	22	12.33	+	1.78	2.01E-05	1.71E-03
regulation of biological process (GO:0050789)	11806	22	13.19	+	1.67	6.52E-05	4.33E-03
biological regulation (GO:0065007)	12228	22	13.66	+	1.61	1.51E-04	8.41E-03
