# Supplementary material for: Genetic analysis of ocular tumour-associated genes using large genomic datasets: insights into selection constraints and variant representation in the population
Source: BMJ Open Ophthalmol. 2024 Feb 21;9(1):e001565. doi: 10.1136/bmjophth-2023-001565 (PMC10897839; doi:10.1136/bmjophth-2023-001565)
Supplement: Supplementary data [file bmjophth-2023-001565supp003.pdf]

Supplementary Table 3: PANTHER Overrepresentation Test (Haploinsufficient Breast Cancer-Associated Genes)

Analysis Type: PANTHER Overrepresentation Test (Released 20221013)							
Annotation Version and Release Date: GO Ontology database DOI: 10.5281/zenodo.7942786 Released 2023-05-10							
Analyzed List: Client Text Box Input (Homo sapiens)							
Reference List: Homo sapiens (all genes in database)							
Test Type: FISHER							
Correction: FDR							
GO biological process complete	Homo sapiens - REFLIST (20589)	Client Text Box Input (6)	Client Text Box Input (expected)	Client Text Box Input (over/under)	Client Text Box Input (fold Enrichment)	Client Text Box Input (raw P-value)	Client Text Box Input (FDR)
anoikis (GO:0043276)	12	3	.00	+	> 100	6.24E-09	9.70E-05
response to UV-B (GO:0010224)	21	2	.01	+	> 100	1.78E-05	1.11E-02
positive regulation of nitric-oxide synthase activity (GO:0051000)	22	2	.01	+	> 100	1.95E-05	1.04E-02
insulin-like growth factor receptor signaling pathway (GO:0048009)	31	2	.01	+	> 100	3.72E-05	1.44E-02
positive regulation of monooxygenase activity (GO:0032770)	32	2	.01	+	> 100	3.95E-05	1.50E-02
negative regulation of macroautophagy (GO:0016242)	32	2	.01	+	> 100	3.95E-05	1.46E-02
response to pain (GO:0048265)	34	2	.01	+	> 100	4.44E-05	1.50E-02
protein localization to chromatin (GO:0071168)	35	2	.01	+	> 100	4.69E-05	1.55E-02
regulation of nitric-oxide synthase activity (GO:0050999)	38	2	.01	+	> 100	5.49E-05	1.61E-02
T cell costimulation (GO:0031295)	40	2	.01	+	> 100	6.06E-05	1.65E-02
lymphocyte costimulation (GO:0031294)	42	2	.01	+	> 100	6.65E-05	1.72E-02

positive regulation of glucose metabolic process (GO:0010907)	42	2	.01	+	> 100	6.65E-05	1.69E-02
positive regulation of nitric oxide biosynthetic process (GO:0045429)	43	2	.01	+	> 100	6.96E-05	1.72E-02
phosphatidylinositol 3-kinase signaling (GO:0014065)	43	2	.01	+	> 100	6.96E-05	1.69E-02
cellular response to vascular endothelial growth factor stimulus (GO:0035924)	45	2	.01	+	> 100	7.60E-05	1.74E-02
positive regulation of nitric oxide metabolic process (GO:1904407)	45	2	.01	+	> 100	7.60E-05	1.71E-02
regulation of extrinsic apoptotic signaling pathway in absence of ligand (GO:2001239)	46	2	.01	+	> 100	7.93E-05	1.73E-02
prostate gland development (GO:0030850)	46	2	.01	+	> 100	7.93E-05	1.71E-02
regulation of monooxygenase activity (GO:0032768)	51	2	.01	+	> 100	9.68E-05	1.95E-02
epidermal growth factor receptor signaling pathway (GO:0007173)	52	2	.02	+	> 100	1.01E-04	1.95E-02
positive regulation of TOR signaling (GO:0032008)	52	2	.02	+	> 100	1.01E-04	1.93E-02
protein kinase B signaling (GO:0043491)	52	2	.02	+	> 100	1.01E-04	1.90E-02
regulation of TORC1 signaling (GO:1903432)	52	2	.02	+	> 100	1.01E-04	1.88E-02
intrinsic apoptotic signaling pathway by p53 class mediator (GO:0072332)	57	2	.02	+	> 100	1.20E-04	2.12E-02
regulation of nitric oxide biosynthetic	58	2	.02	+	> 100	1.24E-04	2.12E-02

process (GO:0045428)							
maternal process involved in female pregnancy (GO:0060135)	61	2	.02	+	> 100	1.37E-04	2.29E-02
mammary gland epithelium development (GO:0061180)	62	2	.02	+	> 100	1.41E-04	2.31E-02
positive regulation of oxidoreductase activity (GO:0051353)	63	2	.02	+	> 100	1.46E-04	2.31E-02
regulation of nitric oxide metabolic process (GO:0080164)	63	2	.02	+	> 100	1.46E-04	2.29E-02
ERBB signaling pathway (GO:0038127)	64	2	.02	+	> 100	1.50E-04	2.34E-02
urogenital system development (GO:0001655)	64	2	.02	+	> 100	1.50E-04	2.31E-02
peptidyl-threonine phosphorylation (GO:0018107)	68	2	.02	+	> 100	1.69E-04	2.50E-02
insulin receptor signaling pathway (GO:0008286)	68	2	.02	+	> 100	1.69E-04	2.48E-02
response to activity (GO:0014823)	70	2	.02	+	98.04	1.79E-04	2.55E-02
positive regulation of peptidyl-serine phosphorylation (GO:0033138)	108	3	.03	+	95.32	3.01E-06	6.68E-03
endothelial cell migration (GO:0043542)	72	2	.02	+	95.32	1.89E-04	2.67E-02
phosphatidylinositol-mediated signaling (GO:0048015)	76	2	.02	+	90.30	2.10E-04	2.87E-02
regulation of TOR signaling (GO:0032006)	116	3	.03	+	88.75	3.71E-06	7.21E-03
peptidyl-threonine modification (GO:0018210)	78	2	.02	+	87.99	2.21E-04	2.99E-02
positive regulation of carbohydrate	78	2	.02	+	87.99	2.21E-04	2.96E-02

metabolic process (GO:0045913)							
inositol lipid-mediated signaling (GO:0048017)	80	2	.02	+	85.79	2.32E-04	3.01E-02
protein localization to chromosome (GO:0034502)	81	2	.02	+	84.73	2.38E-04	3.03E-02
multicellular organismal response to stress (GO:0033555)	83	2	.02	+	82.69	2.50E-04	3.15E-02
positive regulation of smooth muscle cell proliferation (GO:0048661)	85	2	.02	+	80.74	2.62E-04	3.23E-02
negative regulation of autophagy (GO:0010507)	87	2	.03	+	78.89	2.74E-04	3.35E-02
signal transduction by p53 class mediator (GO:0072331)	91	2	.03	+	75.42	2.99E-04	3.55E-02
regulation of peptidyl-serine phosphorylation (GO:0033135)	140	3	.04	+	73.53	6.46E-06	1.11E-02
positive regulation of protein localization to nucleus (GO:1900182)	94	2	.03	+	73.01	3.19E-04	3.67E-02
regulation of carbohydrate biosynthetic process (GO:0043255)	98	2	.03	+	70.03	3.46E-04	3.75E-02
response to UV (GO:0009411)	153	3	.04	+	67.28	8.39E-06	1.18E-02
regulation of glucose metabolic process (GO:0010906)	102	2	.03	+	67.28	3.74E-04	3.95E-02
epithelial cell migration (GO:0010631)	102	2	.03	+	67.28	3.74E-04	3.92E-02
regulation of oxidoreductase activity (GO:0051341)	103	2	.03	+	66.63	3.81E-04	3.97E-02
regulation of generation of precursor	104	2	.03	+	65.99	3.88E-04	4.02E-02

metabolites and energy (GO:0043467)							
epithelium migration (GO:0090132)	105	2	.03	+	65.36	3.96E-04	4.04E-02
regulation of signal transduction by p53 class mediator (GO:1901796)	107	2	.03	+	64.14	4.11E-04	4.14E-02
regulation of protein kinase B signaling (GO:0051896)	167	3	.05	+	61.64	1.09E-05	1.12E-02
tissue migration (GO:0090130)	112	2	.03	+	61.28	4.49E-04	4.44E-02
positive regulation of protein kinase B signaling (GO:0051897)	113	2	.03	+	60.73	4.57E-04	4.43E-02
T cell receptor signaling pathway (GO:0050852)	113	2	.03	+	60.73	4.57E-04	4.41E-02
glucose metabolic process (GO:0006006)	120	2	.03	+	57.19	5.14E-04	4.84E-02
negative regulation of neuron death (GO:1901215)	195	3	.06	+	52.79	1.71E-05	1.16E-02
ameboidal-type cell migration (GO:0001667)	200	3	.06	+	51.47	1.85E-05	1.10E-02
glucose homeostasis (GO:0042593)	203	3	.06	+	50.71	1.93E-05	1.07E-02
carbohydrate homeostasis (GO:0033500)	204	3	.06	+	50.46	1.96E-05	1.01E-02
regulation of neuron death (GO:1901214)	292	3	.09	+	35.26	5.64E-05	1.62E-02
reproductive structure development (GO:0048608)	301	3	.09	+	34.20	6.16E-05	1.65E-02
reproductive system development (GO:0061458)	305	3	.09	+	33.75	6.41E-05	1.69E-02
response to light stimulus (GO:0009416)	316	3	.09	+	32.58	7.11E-05	1.67E-02
gland development (GO:0048732)	425	4	.12	+	32.30	2.69E-06	8.37E-03
positive regulation of lymphocyte	330	3	.10	+	31.20	8.08E-05	1.72E-02

activation (GO:0051251)							
regulation of autophagy (GO:0010506)	352	3	.10	+	29.25	9.77E-05	1.95E-02
positive regulation of cell projection organization (GO:0031346)	354	3	.10	+	29.08	9.94E-05	1.95E-02
response to peptide hormone (GO:0043434)	373	3	.11	+	27.60	1.16E-04	2.07E-02
positive regulation of leukocyte activation (GO:0002696)	379	3	.11	+	27.16	1.21E-04	2.10E-02
positive regulation of cell activation (GO:0050867)	396	3	.12	+	26.00	1.38E-04	2.28E-02
transmembrane receptor protein tyrosine kinase signaling pathway (GO:0007169)	423	3	.12	+	24.34	1.68E-04	2.51E-02
positive regulation of kinase activity (GO:0033674)	430	3	.13	+	23.94	1.76E-04	2.53E-02
response to radiation (GO:0009314)	445	3	.13	+	23.13	1.95E-04	2.72E-02
regulation of neuron projection development (GO:0010975)	450	3	.13	+	22.88	2.01E-04	2.79E-02
response to peptide (GO:1901652)	454	3	.13	+	22.68	2.07E-04	2.84E-02
regulation of plasma membrane bounded cell projection organization (GO:0120035)	643	4	.19	+	21.35	1.38E-05	1.07E-02
positive regulation of cell adhesion (GO:0045785)	486	3	.14	+	21.18	2.52E-04	3.16E-02
regulation of cell projection organization (GO:0031344)	657	4	.19	+	20.89	1.50E-05	1.06E-02
regulation of lymphocyte	504	3	.15	+	20.43	2.81E-04	3.40E-02

activation (GO:0051249)							
cellular response to hormone stimulus (GO:0032870)	510	3	.15	+	20.19	2.91E-04	3.47E-02
response to lipid (GO:0033993)	854	5	.25	+	20.09	7.23E-07	5.61E-03
tube morphogenesis (GO:0035239)	684	4	.20	+	20.07	1.76E-05	1.14E-02
positive regulation of transferase activity (GO:0051347)	523	3	.15	+	19.68	3.13E-04	3.65E-02
negative regulation of intracellular signal transduction (GO:1902532)	524	3	.15	+	19.65	3.15E-04	3.65E-02
cellular response to lipid (GO:0071396)	527	3	.15	+	19.53	3.20E-04	3.65E-02
positive regulation of protein phosphorylation (GO:0001934)	711	4	.21	+	19.31	2.04E-05	1.02E-02
regulation of immune response (GO:0050776)	889	5	.26	+	19.30	8.82E-07	4.57E-03
vasculature development (GO:0001944)	546	3	.16	+	18.85	3.55E-04	3.83E-02
protein localization to organelle (GO:0033365)	731	4	.21	+	18.78	2.28E-05	1.07E-02
positive regulation of hydrolase activity (GO:0051345)	571	3	.17	+	18.03	4.05E-04	4.11E-02
positive regulation of phosphorylation (GO:0042327)	771	4	.22	+	17.80	2.81E-05	1.18E-02
response to hormone (GO:0009725)	791	4	.23	+	17.35	3.11E-05	1.24E-02
multicellular organismal-level homeostasis (GO:0048871)	594	3	.17	+	17.33	4.54E-04	4.43E-02
regulation of leukocyte activation (GO:0002694)	596	3	.17	+	17.27	4.59E-04	4.40E-02
regulation of growth (GO:0040008)	624	3	.18	+	16.50	5.24E-04	4.85E-02

positive regulation of phosphorus metabolic process (GO:0010562)	845	4	.25	+	16.24	4.02E-05	1.45E-02
positive regulation of phosphate metabolic process (GO:0045937)	845	4	.25	+	16.24	4.02E-05	1.42E-02
phosphorylation (GO:0016310)	884	4	.26	+	15.53	4.80E-05	1.55E-02
multicellular organismal reproductive process (GO:0048609)	887	4	.26	+	15.47	4.87E-05	1.54E-02
positive regulation of catalytic activity (GO:0043085)	1112	5	.32	+	15.43	2.67E-06	1.04E-02
tube development (GO:0035295)	900	4	.26	+	15.25	5.15E-05	1.54E-02
multicellular organism reproduction (GO:0032504)	930	4	.27	+	14.76	5.86E-05	1.65E-02
positive regulation of protein modification process (GO:0031401)	935	4	.27	+	14.68	5.98E-05	1.66E-02
developmental process involved in reproduction (GO:0003006)	967	4	.28	+	14.19	6.83E-05	1.71E-02
positive regulation of immune system process (GO:0002684)	977	4	.28	+	14.05	7.11E-05	1.70E-02
apoptotic process (GO:0006915)	1047	4	.31	+	13.11	9.31E-05	1.90E-02
regulation of protein phosphorylation (GO:0001932)	1071	4	.31	+	12.82	1.02E-04	1.88E-02
programmed cell death (GO:0012501)	1093	4	.32	+	12.56	1.10E-04	2.01E-02
cell death (GO:0008219)	1105	4	.32	+	12.42	1.15E-04	2.08E-02
response to abiotic stimulus (GO:0009628)	1118	4	.33	+	12.28	1.20E-04	2.10E-02
cellular response to oxygen-containing compound (GO:1901701)	1146	4	.33	+	11.98	1.32E-04	2.24E-02

regulation of phosphorylation (GO:0042325)	1170	4	.34	+	11.73	1.44E-04	2.32E-02
positive regulation of gene expression (GO:0010628)	1173	4	.34	+	11.70	1.45E-04	2.32E-02
regulation of apoptotic process (GO:0042981)	1471	5	.43	+	11.66	1.06E-05	1.18E-02
regulation of immune system process (GO:0002682)	1488	5	.43	+	11.53	1.12E-05	1.09E-02
positive regulation of molecular function (GO:0044093)	1519	5	.44	+	11.30	1.24E-05	1.07E-02
regulation of programmed cell death (GO:0043067)	1521	5	.44	+	11.28	1.25E-05	1.02E-02
intracellular signal transduction (GO:0035556)	1558	5	.45	+	11.01	1.41E-05	1.04E-02
regulation of phosphate metabolic process (GO:0019220)	1315	4	.38	+	10.44	2.26E-04	3.00E-02
regulation of phosphorus metabolic process (GO:0051174)	1316	4	.38	+	10.43	2.27E-04	2.99E-02
positive regulation of developmental process (GO:0051094)	1319	4	.38	+	10.41	2.29E-04	2.99E-02
positive regulation of multicellular organismal process (GO:0051240)	1654	5	.48	+	10.37	1.89E-05	1.09E-02
response to oxygen-containing compound (GO:1901700)	1681	5	.49	+	10.21	2.04E-05	9.92E-03
regulation of intracellular signal transduction (GO:1902531)	1727	5	.50	+	9.93	2.34E-05	1.07E-02
positive regulation of protein metabolic process (GO:0051247)	1425	4	.42	+	9.63	3.09E-04	3.64E-02

regulation of protein modification process (GO:0031399)	1443	4	.42	+	9.51	3.24E-04	3.68E-02
reproductive process (GO:0022414)	1448	4	.42	+	9.48	3.29E-04	3.70E-02
reproduction (GO:0000003)	1458	4	.42	+	9.41	3.38E-04	3.77E-02
regulation of catalytic activity (GO:0050790)	1827	5	.53	+	9.39	3.08E-05	1.26E-02
response to endogenous stimulus (GO:0009719)	1464	4	.43	+	9.38	3.43E-04	3.75E-02
positive regulation of signal transduction (GO:0009967)	1549	4	.45	+	8.86	4.27E-04	4.25E-02
cellular response to stress (GO:0033554)	1603	4	.47	+	8.56	4.87E-04	4.64E-02
regulation of developmental process (GO:0050793)	2462	6	.72	+	8.36	2.95E-06	7.62E-03
cell development (GO:0048468)	2185	5	.64	+	7.85	7.41E-05	1.72E-02
positive regulation of response to stimulus (GO:0048584)	2203	5	.64	+	7.79	7.71E-05	1.71E-02
anatomical structure morphogenesis (GO:0009653)	2244	5	.65	+	7.65	8.44E-05	1.77E-02
immune system process (GO:0002376)	2277	5	.66	+	7.54	9.06E-05	1.88E-02
animal organ development (GO:0048513)	2925	6	.85	+	7.04	8.27E-06	1.28E-02
positive regulation of cellular metabolic process (GO:0031325)	2976	6	.87	+	6.92	9.17E-06	1.19E-02
regulation of multicellular organismal process (GO:0051239)	2983	6	.87	+	6.90	9.31E-06	1.11E-02
regulation of molecular function (GO:0065009)	2569	5	.75	+	6.68	1.63E-04	2.49E-02

response to organic substance (GO:0010033)	2592	5	.76	+	6.62	1.71E-04	2.48E-02
positive regulation of nitrogen compound metabolic process (GO:0051173)	3121	6	.91	+	6.60	1.22E-05	1.11E-02
positive regulation of macromolecule metabolic process (GO:0010604)	3514	6	1.02	+	5.86	2.48E-05	1.10E-02
system development (GO:0048731)	3553	6	1.04	+	5.79	2.65E-05	1.14E-02
negative regulation of metabolic process (GO:0009892)	2985	5	.87	+	5.75	3.39E-04	3.76E-02
regulation of signal transduction (GO:0009966)	2992	5	.87	+	5.73	3.43E-04	3.78E-02
positive regulation of metabolic process (GO:0009893)	3825	6	1.11	+	5.38	4.13E-05	1.43E-02
regulation of response to stimulus (GO:0048583)	3960	6	1.15	+	5.20	5.08E-05	1.58E-02
multicellular organism development (GO:0007275)	3966	6	1.16	+	5.19	5.13E-05	1.56E-02
signal transduction (GO:0007165)	4819	6	1.40	+	4.27	1.65E-04	2.49E-02
signaling (GO:0023052)	5119	6	1.49	+	4.02	2.37E-04	3.04E-02
anatomical structure development (GO:0048856)	5201	6	1.52	+	3.96	2.61E-04	3.24E-02
cell communication (GO:0007154)	5278	6	1.54	+	3.90	2.85E-04	3.43E-02
regulation of cellular metabolic process (GO:0031323)	5482	6	1.60	+	3.76	3.57E-04	3.83E-02
positive regulation of cellular process (GO:0048522)	5511	6	1.61	+	3.74	3.69E-04	3.92E-02
cellular component organization (GO:0016043)	5560	6	1.62	+	3.70	3.89E-04	4.00E-02
regulation of nitrogen compound	5646	6	1.65	+	3.65	4.26E-04	4.27E-02

metabolic process (GO:0051171)							
developmental process (GO:0032502)	5702	6	1.66	+	3.61	4.52E-04	4.45E-02
cellular component organization or biogenesis (GO:0071840)	5774	6	1.68	+	3.57	4.88E-04	4.62E-02
regulation of primary metabolic process (GO:0080090)	5825	6	1.70	+	3.53	5.14E-04	4.81E-02
cellular metabolic process (GO:0044237)	5828	6	1.70	+	3.53	5.16E-04	4.80E-02
