# Supplementary material for: Genetic analysis of ocular tumour-associated genes using large genomic datasets: insights into selection constraints and variant representation in the population
Source: BMJ Open Ophthalmol. 2024 Feb 21;9(1):e001565. doi: 10.1136/bmjophth-2023-001565 (PMC10897839; doi:10.1136/bmjophth-2023-001565)
Supplement: Supplementary data [file bmjophth-2023-001565supp004.pdf]

Supplementary Table 4: PANTHER Overrepresentation Test (Haploinsufficient Lung Cancer-Associated Genes)

Analysis Type:				PANTHER Overrepresentation Test (Released 20231017)			
Annotation Version and Release Date:				GO Ontology database DOI: 10.5281/zenodo.10536401 Released 2024-01-17			
Analyzed List:				Client Text Box Input (Homo sapiens)			
Reference List:				Homo sapiens (all genes in database)			
Test Type:				FISHER			
Correction:				FDR			
GO biological process complete	Homo sapiens - REFLIST (20592)	Client Text Box Input (28)	Client Text Box Input (expected)	Client Text Box Input (over/under)	Client Text Box Input (fold Enrichment)	Client Text Box Input (raw P-value)	Client Text Box Input (FDR)
regulation of Golgi inheritance (GO:0090170)	4	2	.01	+	> 100	2.66E-05	1.45E-03
trachea formation (GO:0060440)	7	3	.01	+	> 100	2.67E-07	2.81E-05
trachea morphogenesis (GO:0060439)	11	3	.01	+	> 100	8.08E-07	7.33E-05
Bergmann glial cell differentiation (GO:0060020)	11	3	.01	+	> 100	8.08E-07	7.29E-05
anoikis (GO:0043276)	12	3	.02	+	> 100	1.01E-06	8.94E-05
ERBB2-ERBB3 signaling pathway (GO:0038133)	9	2	.01	+	> 100	9.71E-05	4.11E-03
ERBB3 signaling pathway (GO:0038129)	9	2	.01	+	> 100	9.71E-05	4.10E-03
epithelial cell proliferation involved in prostate gland development (GO:0060767)	9	2	.01	+	> 100	9.71E-05	4.09E-03
regulation of epithelial cell proliferation involved in prostate gland development (GO:0060768)	10	2	.01	+	> 100	1.16E-04	4.81E-03
negative regulation of fibroblast	11	2	.01	+	> 100	1.37E-04	5.48E-03

migration (GO:0010764)							
genitalia morphogenesis (GO:0035112)	12	2	.02	+	> 100	1.60E-04	6.26E-03
trachea development (GO:0060438)	19	3	.03	+	> 100	3.39E-06	2.59E-04
nucleosome disassembly (GO:0006337)	19	3	.03	+	> 100	3.39E-06	2.57E-04
myoblast proliferation (GO:0051450)	13	2	.02	+	> 100	1.85E-04	7.09E-03
protein-DNA complex disassembly (GO:0032986)	21	3	.03	+	> 100	4.45E-06	3.23E-04
layer formation in cerebral cortex (GO:0021819)	15	2	.02	+	98.06	2.39E-04	8.92E-03
thyroid gland development (GO:0030878)	31	4	.04	+	94.89	1.38E-07	1.62E-05
insulin-like growth factor receptor signaling pathway (GO:0048009)	32	4	.04	+	91.93	1.55E-07	1.74E-05
regulation of Golgi organization (GO:1903358)	16	2	.02	+	91.93	2.68E-04	9.70E-03
thymus development (GO:0048538)	50	6	.07	+	88.25	1.10E-10	4.07E-08
positive regulation of nitric-oxide synthase activity (GO:0051000)	17	2	.02	+	86.52	3.00E-04	1.07E-02
regulation of nucleotide-excision repair (GO:2000819)	28	3	.04	+	78.80	9.83E-06	6.43E-04
regulation of oxidative stress-induced neuron intrinsic apoptotic signaling pathway (GO:1903376)	19	2	.03	+	77.41	3.67E-04	1.25E-02
ERK1 and ERK2 cascade (GO:0070371)	49	5	.07	+	75.04	9.56E-09	1.68E-06
regulation of early endosome to late	20	2	.03	+	73.54	4.04E-04	1.34E-02

endosome transport (GO:2000641)							
animal organ formation (GO:0048645)	40	4	.05	+	73.54	3.56E -07	3.64E -05
stress fiber assembly (GO:0043149)	20	2	.03	+	73.54	4.04E -04	1.34E -02
labyrinthine layer blood vessel development (GO:0060716)	20	2	.03	+	73.54	4.04E -04	1.34E -02
contractile actin filament bundle assembly (GO:0030038)	20	2	.03	+	73.54	4.04E -04	1.33E -02
negative regulation of endothelial cell apoptotic process (GO:2000352)	31	3	.04	+	71.17	1.30E -05	8.15E -04
placenta blood vessel development (GO:0060674)	31	3	.04	+	71.17	1.30E -05	8.12E -04
ERBB signaling pathway (GO:0038127)	65	6	.09	+	67.89	4.77E -10	1.30E -07
positive regulation of myoblast differentiation (GO:0045663)	44	4	.06	+	66.86	5.08E -07	4.93E -05
organ induction (GO:0001759)	22	2	.03	+	66.86	4.82E -04	1.55E -02
regulation of extrinsic apoptotic signaling pathway in absence of ligand (GO:2001239)	46	4	.06	+	63.95	6.00E -07	5.72E -05
ERBB2 signaling pathway (GO:0038128)	23	2	.03	+	63.95	5.23E -04	1.66E -02
negative regulation of cellular senescence (GO:2000773)	24	2	.03	+	61.29	5.66E -04	1.77E -02
male genitalia development (GO:0030539)	24	2	.03	+	61.29	5.66E -04	1.76E -02
negative regulation of extrinsic apoptotic signaling pathway in absence of ligand (GO:2001240)	36	3	.05	+	61.29	1.98E -05	1.15E -03

regulation of G0 to G1 transition (GO:0070316)	36	3	.05	+	61.29	1.98E-05	1.15E-03
negative regulation of signal transduction in absence of ligand (GO:1901099)	36	3	.05	+	61.29	1.98E-05	1.15E-03
epidermal growth factor receptor signaling pathway (GO:0007173)	49	4	.07	+	60.03	7.61E-07	6.94E-05
lung morphogenesis (GO:0060425)	50	4	.07	+	58.83	8.21E-07	7.36E-05
cardiac muscle cell proliferation (GO:0060038)	25	2	.03	+	58.83	6.11E-04	1.87E-02
response to muscle stretch (GO:0035994)	25	2	.03	+	58.83	6.11E-04	1.86E-02
androgen receptor signaling pathway (GO:0030521)	25	2	.03	+	58.83	6.11E-04	1.86E-02
regulation of fibroblast migration (GO:0010762)	38	3	.05	+	58.06	2.31E-05	1.28E-03
lung epithelium development (GO:0060428)	39	3	.05	+	56.57	2.48E-05	1.37E-03
lung epithelial cell differentiation (GO:0060487)	26	2	.04	+	56.57	6.58E-04	1.98E-02
lung cell differentiation (GO:0060479)	26	2	.04	+	56.57	6.58E-04	1.97E-02
regulation of nitric-oxide synthase activity (GO:0050999)	26	2	.04	+	56.57	6.58E-04	1.97E-02
metanephros morphogenesis (GO:0003338)	26	2	.04	+	56.57	6.58E-04	1.97E-02
epithelial tube branching involved in lung morphogenesis (GO:0060441)	27	2	.04	+	54.48	7.06E-04	2.09E-02
regulation of oxidative stress-induced intrinsic apoptotic signaling	42	3	.06	+	52.53	3.06E-05	1.64E-03

pathway (GO:1902175)							
telencephalon glial cell migration (GO:0022030)	28	2	.04	+	52.53	7.56E -04	2.20E -02
positive regulation of monooxygenase activity (GO:0032770)	28	2	.04	+	52.53	7.56E -04	2.20E -02
positive regulation of protein tyrosine kinase activity (GO:0061098)	28	2	.04	+	52.53	7.56E -04	2.19E -02
cerebral cortex radial glia-guided migration (GO:0021801)	28	2	.04	+	52.53	7.56E -04	2.19E -02
Schwann cell development (GO:0014044)	43	3	.06	+	51.31	3.27E -05	1.71E -03
positive regulation of oxidoreductase activity (GO:0051353)	43	3	.06	+	51.31	3.27E -05	1.71E -03
negative regulation of oxidative stress-induced intrinsic apoptotic signaling pathway (GO:1902176)	29	2	.04	+	50.72	8.07E -04	2.29E -02
regulation of axon regeneration (GO:0048679)	29	2	.04	+	50.72	8.07E -04	2.29E -02
regulation of animal organ formation (GO:0003156)	29	2	.04	+	50.72	8.07E -04	2.29E -02
positive regulation of G1/S transition of mitotic cell cycle (GO:1900087)	44	3	.06	+	50.14	3.49E -05	1.78E -03
positive regulation of glucose transmembrane transport (GO:0010828)	44	3	.06	+	50.14	3.49E -05	1.77E -03
sister chromatid cohesion (GO:0007062)	45	3	.06	+	49.03	3.72E -05	1.89E -03
positive regulation of extracellular	30	2	.04	+	49.03	8.60E -04	2.42E -02

matrix organization (GO:1903055)							
positive regulation of signaling receptor activity (GO:2000273)	30	2	.04	+	49.03	8.60E -04	2.41E -02
negative regulation of epithelial cell apoptotic process (GO:1904036)	61	4	.08	+	48.22	1.74E -06	1.45E -04
positive regulation of transforming growth factor beta receptor signaling pathway (GO:0030511)	31	2	.04	+	47.45	9.15E -04	2.54E -02
positive regulation of cellular response to transforming growth factor beta stimulus (GO:1903846)	31	2	.04	+	47.45	9.15E -04	2.54E -02
prostate gland development (GO:0030850)	47	3	.06	+	46.94	4.21E -05	2.08E -03
labyrinthine layer development (GO:0060711)	47	3	.06	+	46.94	4.21E -05	2.08E -03
positive regulation of stem cell proliferation (GO:2000648)	48	3	.07	+	45.96	4.47E -05	2.18E -03
Schwann cell differentiation (GO:0014037)	48	3	.07	+	45.96	4.47E -05	2.17E -03
T cell differentiation in thymus (GO:0033077)	64	4	.09	+	45.96	2.09E -06	1.69E -04
regulation of neuron projection regeneration (GO:0070570)	32	2	.04	+	45.96	9.71E -04	2.66E -02
protein localization to cell surface (GO:0034394)	32	2	.04	+	45.96	9.71E -04	2.65E -02
regulation of mitophagy (GO:1901524)	33	2	.04	+	44.57	1.03E -03	2.80E -02
astrocyte differentiation (GO:0048708)	66	4	.09	+	44.57	2.34E -06	1.87E -04

developmental induction (GO:0031128)	33	2	.04	+	44.57	1.03E-03	2.79E-02
hair follicle morphogenesis (GO:0031069)	33	2	.04	+	44.57	1.03E-03	2.79E-02
negative regulation of macroautophagy (GO:0016242)	33	2	.04	+	44.57	1.03E-03	2.78E-02
long-term synaptic potentiation (GO:0060291)	50	3	.07	+	44.13	5.02E-05	2.41E-03
hematopoietic or lymphoid organ development (GO:0048534)	100	6	.14	+	44.13	5.50E-09	1.06E-06
negative regulation of osteoclast differentiation (GO:0045671)	34	2	.05	+	43.26	1.09E-03	2.91E-02
lipopolysaccharide-mediated signaling pathway (GO:0031663)	34	2	.05	+	43.26	1.09E-03	2.90E-02
cardiac muscle tissue growth (GO:0055017)	34	2	.05	+	43.26	1.09E-03	2.90E-02
regulation of endothelial cell apoptotic process (GO:2000351)	52	3	.07	+	42.43	5.61E-05	2.64E-03
regulation of stress-activated MAPK cascade (GO:0032872)	35	2	.05	+	42.02	1.15E-03	3.01E-02
proximal/distal pattern formation (GO:0009954)	35	2	.05	+	42.02	1.15E-03	3.01E-02
positive regulation of telomere maintenance via telomerase (GO:0032212)	35	2	.05	+	42.02	1.15E-03	3.00E-02
positive T cell selection (GO:0043368)	35	2	.05	+	42.02	1.15E-03	3.00E-02
face development (GO:0060324)	53	3	.07	+	41.63	5.92E-05	2.76E-03
regulation of morphogenesis of a	54	3	.07	+	40.86	6.25E-05	2.88E-03

branching structure (GO:0060688)							
heart growth (GO:0060419)	36	2	.05	+	40.86	1.21E -03	3.11E -02
positive regulation of animal organ morphogenesis (GO:0110110)	36	2	.05	+	40.86	1.21E -03	3.11E -02
smooth muscle cell differentiation (GO:0051145)	36	2	.05	+	40.86	1.21E -03	3.10E -02
response to pain (GO:0048265)	36	2	.05	+	40.86	1.21E -03	3.10E -02
regulation of phospholipase C activity (GO:1900274)	36	2	.05	+	40.86	1.21E -03	3.09E -02
striated muscle cell proliferation (GO:0014855)	36	2	.05	+	40.86	1.21E -03	3.09E -02
positive regulation of glucose import (GO:0046326)	36	2	.05	+	40.86	1.21E -03	3.08E -02
endothelial cell migration (GO:0043542)	73	4	.10	+	40.30	3.44E -06	2.58E -04
peptidyl-threonine phosphorylation (GO:0018107)	55	3	.07	+	40.11	6.58E -05	3.02E -03
cerebral cortex radially oriented cell migration (GO:0021799)	37	2	.05	+	39.75	1.28E -03	3.23E -02
epidermis morphogenesis (GO:0048730)	37	2	.05	+	39.75	1.28E -03	3.23E -02
positive regulation of morphogenesis of an epithelium (GO:1905332)	37	2	.05	+	39.75	1.28E -03	3.22E -02
regulation of myoblast differentiation (GO:0045661)	75	4	.10	+	39.22	3.81E -06	2.81E -04
epithelial cell apoptotic process (GO:1904019)	57	3	.08	+	38.71	7.29E -05	3.25E -03
endothelial cell proliferation (GO:0001935)	38	2	.05	+	38.71	1.34E -03	3.36E -02

cellular response to cadmium ion (GO:0071276)	38	2	.05	+	38.71	1.34E -03	3.36E -02
regulation of ubiquitin-protein transferase activity (GO:0051438)	38	2	.05	+	38.71	1.34E -03	3.35E -02
spleen development (GO:0048536)	38	2	.05	+	38.71	1.34E -03	3.35E -02
positive regulation of telomere maintenance via telomere lengthening (GO:1904358)	38	2	.05	+	38.71	1.34E -03	3.34E -02
transcription initiation-coupled chromatin remodeling (GO:0045815)	38	2	.05	+	38.71	1.34E -03	3.34E -02
negative regulation of Notch signaling pathway (GO:0045746)	39	2	.05	+	37.71	1.41E -03	3.47E -02
regulation of monooxygenase activity (GO:0032768)	39	2	.05	+	37.71	1.41E -03	3.47E -02
regulation of cell migration involved in sprouting angiogenesis (GO:0090049)	39	2	.05	+	37.71	1.41E -03	3.46E -02
regulation of stress-activated protein kinase signaling cascade (GO:0070302)	39	2	.05	+	37.71	1.41E -03	3.46E -02
positive regulation of cell cycle G1/S phase transition (GO:1902808)	59	3	.08	+	37.39	8.04E -05	3.52E -03
positive regulation of gene expression, epigenetic (GO:0141137)	40	2	.05	+	36.77	1.48E -03	3.61E -02
response to cadmium ion (GO:0046686)	61	3	.08	+	36.17	8.84E -05	3.81E -03
mammary gland epithelium	61	3	.08	+	36.17	8.84E -05	3.79E -03

development (GO:0061180)							
regulation of autophagy of mitochondrion (GO:1903146)	41	2	.06	+	35.87	1.55E -03	3.75E -02
regulation of protein localization to cell surface (GO:2000008)	41	2	.06	+	35.87	1.55E -03	3.75E -02
positive regulation of glucose metabolic process (GO:0010907)	41	2	.06	+	35.87	1.55E -03	3.74E -02
negative regulation of gliogenesis (GO:0014014)	42	2	.06	+	35.02	1.62E -03	3.91E -02
urogenital system development (GO:0001655)	64	3	.09	+	34.47	1.01E -04	4.24E -03
T cell costimulation (GO:0031295)	43	2	.06	+	34.21	1.70E -03	4.08E -02
positive regulation of substrate adhesion-dependent cell spreading (GO:1900026)	43	2	.06	+	34.21	1.70E -03	4.07E -02
peptidyl-threonine modification (GO:0018210)	65	3	.09	+	33.94	1.06E -04	4.42E -03
regulation of morphogenesis of an epithelium (GO:1905330)	65	3	.09	+	33.94	1.06E -04	4.41E -03
muscle cell proliferation (GO:0033002)	44	2	.06	+	33.43	1.77E -03	4.24E -02
lymphocyte costimulation (GO:0031294)	44	2	.06	+	33.43	1.77E -03	4.23E -02
T cell homeostasis (GO:0043029)	44	2	.06	+	33.43	1.77E -03	4.22E -02
cerebellum morphogenesis (GO:0021587)	45	2	.06	+	32.69	1.85E -03	4.39E -02
cellular response to vascular endothelial growth factor stimulus (GO:0035924)	45	2	.06	+	32.69	1.85E -03	4.38E -02

regulation of mitotic metaphase/anaphase transition (GO:0030071)	90	4	.12	+	32.69	7.62E-06	5.21E-04
positive regulation of mitotic cell cycle phase transition (GO:1901992)	91	4	.12	+	32.33	7.95E-06	5.39E-04
regulation of oxidoreductase activity (GO:0051341)	69	3	.09	+	31.98	1.26E-04	5.16E-03
positive regulation of neuron apoptotic process (GO:0043525)	69	3	.09	+	31.98	1.26E-04	5.15E-03
insulin receptor signaling pathway (GO:0008286)	69	3	.09	+	31.98	1.26E-04	5.13E-03
regulation of cellular senescence (GO:2000772)	46	2	.06	+	31.98	1.93E-03	4.55E-02
response to epidermal growth factor (GO:0070849)	46	2	.06	+	31.98	1.93E-03	4.55E-02
regulation of myelination (GO:0031641)	46	2	.06	+	31.98	1.93E-03	4.54E-02
endodermal cell differentiation (GO:0035987)	46	2	.06	+	31.98	1.93E-03	4.53E-02
regulation of long-term synaptic potentiation (GO:1900271)	46	2	.06	+	31.98	1.93E-03	4.53E-02
substrate adhesion-dependent cell spreading (GO:0034446)	46	2	.06	+	31.98	1.93E-03	4.52E-02
cellular response to dopamine (GO:1903351)	46	2	.06	+	31.98	1.93E-03	4.51E-02
T cell receptor signaling pathway (GO:0050852)	116	5	.16	+	31.70	5.65E-07	5.41E-05
regulation of metaphase/anaphase transition of cell cycle (GO:1902099)	93	4	.13	+	31.63	8.64E-06	5.78E-04

lymphocyte homeostasis (GO:0002260)	70	3	.10	+	31.52	1.31E -04	5.25E -03
pancreas development (GO:0031016)	70	3	.10	+	31.52	1.31E -04	5.24E -03
regulation of G1/S transition of mitotic cell cycle (GO:2000045)	164	7	.22	+	31.39	2.44E -09	5.55E -07
regulation of cell cycle G1/S phase transition (GO:1902806)	191	8	.26	+	30.80	1.72E -10	5.70E -08
hindbrain morphogenesis (GO:0021575)	48	2	.07	+	30.64	2.09E -03	4.85E -02
positive regulation of stem cell population maintenance (GO:1902459)	48	2	.07	+	30.64	2.09E -03	4.85E -02
T cell selection (GO:0045058)	48	2	.07	+	30.64	2.09E -03	4.84E -02
genitalia development (GO:0048806)	48	2	.07	+	30.64	2.09E -03	4.83E -02
negative regulation of cold-induced thermogenesis (GO:0120163)	48	2	.07	+	30.64	2.09E -03	4.82E -02
cellular response to nerve growth factor stimulus (GO:1990090)	48	2	.07	+	30.64	2.09E -03	4.82E -02
glial cell migration (GO:0008347)	48	2	.07	+	30.64	2.09E -03	4.81E -02
response to dopamine (GO:1903350)	48	2	.07	+	30.64	2.09E -03	4.80E -02
positive regulation of TOR signaling (GO:0032008)	72	3	.10	+	30.64	1.42E -04	5.64E -03
positive regulation of mitotic cell cycle (GO:0045931)	122	5	.17	+	30.14	7.18E -07	6.64E -05
cerebral cortex cell migration (GO:0021795)	49	2	.07	+	30.02	2.18E -03	4.97E -02
cellular response to catecholamine	49	2	.07	+	30.02	2.18E -03	4.96E -02

stimulus (GO:0071870)							
cellular response to monoamine stimulus (GO:0071868)	49	2	.07	+	30.02	2.18E-03	4.96E-02
canonical NF-kappaB signal transduction (GO:0007249)	49	2	.07	+	30.02	2.18E-03	4.95E-02
digestive tract morphogenesis (GO:0048546)	49	2	.07	+	30.02	2.18E-03	4.94E-02
negative regulation of epithelial cell differentiation (GO:0030857)	49	2	.07	+	30.02	2.18E-03	4.94E-02
regulation of glucose transmembrane transport (GO:0010827)	75	3	.10	+	29.42	1.59E-04	6.24E-03
epithelial cell proliferation (GO:0050673)	178	7	.24	+	28.92	4.22E-09	8.69E-07
regulation of epithelial cell apoptotic process (GO:1904035)	103	4	.14	+	28.56	1.27E-05	8.00E-04
epithelial cell migration (GO:0010631)	104	4	.14	+	28.29	1.32E-05	8.16E-04
regulation of sister chromatid segregation (GO:0033045)	105	4	.14	+	28.02	1.37E-05	8.43E-04
alpha-beta T cell differentiation (GO:0046632)	79	3	.11	+	27.93	1.85E-04	7.08E-03
positive regulation of axonogenesis (GO:0050772)	80	3	.11	+	27.58	1.92E-04	7.32E-03
epithelium migration (GO:0090132)	107	4	.15	+	27.49	1.47E-05	8.99E-04
glial cell differentiation (GO:0010001)	218	8	.30	+	26.99	4.73E-10	1.31E-07
regulation of stem cell proliferation (GO:0072091)	83	3	.11	+	26.58	2.13E-04	8.03E-03
positive regulation of cell cycle phase transition (GO:1901989)	111	4	.15	+	26.50	1.70E-05	1.01E-03

immune system development (GO:0002520)	167	6	.23	+	26.42	1.03E-07	1.29E-05
positive regulation of smooth muscle cell proliferation (GO:0048661)	85	3	.12	+	25.96	2.28E-04	8.55E-03
positive regulation of Wnt signaling pathway (GO:0030177)	142	5	.19	+	25.90	1.48E-06	1.25E-04
tissue migration (GO:0090130)	114	4	.16	+	25.80	1.88E-05	1.10E-03
positive regulation of double-strand break repair (GO:2000781)	86	3	.12	+	25.65	2.35E-04	8.82E-03
embryonic placenta development (GO:0001892)	87	3	.12	+	25.36	2.43E-04	9.01E-03
associative learning (GO:0008306)	88	3	.12	+	25.07	2.51E-04	9.28E-03
negative regulation of protein modification by small protein conjugation or removal (GO:1903321)	88	3	.12	+	25.07	2.51E-04	9.26E-03
positive regulation of binding (GO:0051099)	118	4	.16	+	24.93	2.14E-05	1.22E-03
negative regulation of protein serine/threonine kinase activity (GO:0071901)	89	3	.12	+	24.79	2.60E-04	9.49E-03
peripheral nervous system development (GO:0007422)	90	3	.12	+	24.51	2.68E-04	9.73E-03
negative regulation of autophagy (GO:0010507)	90	3	.12	+	24.51	2.68E-04	9.71E-03
regulation of fibroblast proliferation (GO:0048145)	91	3	.12	+	24.24	2.77E-04	9.97E-03
regulation of T cell differentiation (GO:0045580)	183	6	.25	+	24.11	1.74E-07	1.93E-05

negative regulation of post-translational protein modification (GO:1901874)	92	3	.13	+	23.98	2.85E-04	1.03E-02
regulation of stress fiber assembly (GO:0051492)	92	3	.13	+	23.98	2.85E-04	1.02E-02
regulation of blood vessel endothelial cell migration (GO:0043535)	92	3	.13	+	23.98	2.85E-04	1.02E-02
gliogenesis (GO:0042063)	277	9	.38	+	23.89	9.19E-11	3.59E-08
glial cell development (GO:0021782)	128	4	.17	+	22.98	2.92E-05	1.58E-03
negative regulation of extrinsic apoptotic signaling pathway (GO:2001237)	97	3	.13	+	22.75	3.32E-04	1.17E-02
leukocyte homeostasis (GO:0001776)	97	3	.13	+	22.75	3.32E-04	1.17E-02
positive regulation of protein localization to nucleus (GO:1900182)	97	3	.13	+	22.75	3.32E-04	1.16E-02
antigen receptor-mediated signaling pathway (GO:0050851)	162	5	.22	+	22.70	2.77E-06	2.17E-04
positive regulation of DNA repair (GO:0045739)	130	4	.18	+	22.63	3.09E-05	1.65E-03
regulation of Notch signaling pathway (GO:0008593)	98	3	.13	+	22.51	3.42E-04	1.19E-02
regulation of chromosome segregation (GO:0051983)	131	4	.18	+	22.46	3.18E-05	1.69E-03
regulation of double-strand break repair (GO:2000779)	132	4	.18	+	22.29	3.28E-05	1.71E-03
endocrine system development (GO:0035270)	132	4	.18	+	22.29	3.28E-05	1.70E-03
regulation of dendrite	99	3	.13	+	22.29	3.52E-04	1.21E-02

development (GO:0050773)							
regulation of Wnt signaling pathway (GO:0030111)	336	10	.46	+	21.89	1.56E -11	1.19E -08
regulation of actomyosin structure organization (GO:0110020)	102	3	.14	+	21.63	3.83E -04	1.30E -02
organ growth (GO:0035265)	102	3	.14	+	21.63	3.83E -04	1.30E -02
regulation of actin filament bundle assembly (GO:0032231)	104	3	.14	+	21.21	4.05E -04	1.33E -02
oogenesis (GO:0048477)	104	3	.14	+	21.21	4.05E -04	1.33E -02
regulation of smooth muscle cell proliferation (GO:0048660)	139	4	.19	+	21.16	3.99E -05	1.99E -03
positive regulation of endothelial cell migration (GO:0010595)	106	3	.14	+	20.81	4.28E -04	1.40E -02
MAPK cascade (GO:0000165)	213	6	.29	+	20.72	4.15E -07	4.17E -05
regulation of TOR signaling (GO:0032006)	143	4	.19	+	20.57	4.44E -05	2.17E -03
regulation of lymphocyte differentiation (GO:0045619)	215	6	.29	+	20.52	4.38E -07	4.34E -05
nucleosome organization (GO:0034728)	108	3	.15	+	20.43	4.51E -04	1.46E -02
regulation of leukocyte differentiation (GO:1902105)	327	9	.44	+	20.24	3.83E -10	1.08E -07
alpha-beta T cell activation (GO:0046631)	109	3	.15	+	20.24	4.63E -04	1.49E -02
T cell differentiation (GO:0030217)	184	5	.25	+	19.98	5.08E -06	3.64E -04
cellular response to insulin stimulus (GO:0032869)	149	4	.20	+	19.74	5.19E -05	2.48E -03
negative regulation of leukocyte	112	3	.15	+	19.70	5.00E -04	1.60E -02

differentiation (GO:1902106)							
developmental cell growth (GO:0048588)	112	3	.15	+	19.70	5.00E-04	1.60E-02
positive regulation of epithelial cell migration (GO:0010634)	151	4	.21	+	19.48	5.46E-05	2.59E-03
lung development (GO:0030324)	189	5	.26	+	19.46	5.77E-06	4.11E-04
regulation of circadian rhythm (GO:0042752)	114	3	.16	+	19.35	5.26E-04	1.67E-02
cell growth (GO:0016049)	114	3	.16	+	19.35	5.26E-04	1.66E-02
epigenetic regulation of gene expression (GO:0040029)	192	5	.26	+	19.15	6.22E-06	4.39E-04
respiratory tube development (GO:0030323)	193	5	.26	+	19.05	6.37E-06	4.48E-04
regulation of extrinsic apoptotic signaling pathway (GO:2001236)	155	4	.21	+	18.98	6.03E-05	2.80E-03
negative regulation of apoptotic signaling pathway (GO:2001234)	234	6	.32	+	18.86	7.10E-07	6.60E-05
negative regulation of hemopoiesis (GO:1903707)	117	3	.16	+	18.86	5.66E-04	1.77E-02
stem cell population maintenance (GO:0019827)	117	3	.16	+	18.86	5.66E-04	1.77E-02
positive regulation of T cell differentiation (GO:0045582)	119	3	.16	+	18.54	5.94E-04	1.82E-02
female gamete generation (GO:0007292)	159	4	.22	+	18.50	6.65E-05	3.04E-03
skin epidermis development (GO:0098773)	120	3	.16	+	18.39	6.08E-04	1.86E-02
maintenance of cell number (GO:0098727)	121	3	.16	+	18.23	6.23E-04	1.88E-02

regulation of neuron apoptotic process (GO:0043523)	243	6	.33	+	18.16	8.80E-07	7.85E-05
negative regulation of neuron apoptotic process (GO:0043524)	162	4	.22	+	18.16	7.13E-05	3.19E-03
negative regulation of protein kinase activity (GO:0006469)	164	4	.22	+	17.94	7.47E-05	3.30E-03
positive regulation of peptidyl-tyrosine phosphorylation (GO:0050731)	164	4	.22	+	17.94	7.47E-05	3.29E-03
regulation of mononuclear cell migration (GO:0071675)	124	3	.17	+	17.79	6.68E-04	1.99E-02
regulation of chromosome organization (GO:0033044)	249	6	.34	+	17.72	1.01E-06	8.91E-05
regulation of endothelial cell migration (GO:0010594)	168	4	.23	+	17.51	8.19E-05	3.58E-03
positive regulation of T cell activation (GO:0050870)	253	6	.34	+	17.44	1.11E-06	9.59E-05
positive regulation of ERK1 and ERK2 cascade (GO:0070374)	211	5	.29	+	17.43	9.72E-06	6.39E-04
regulation of T cell activation (GO:0050863)	380	9	.52	+	17.42	1.39E-09	3.31E-07
positive regulation of transmembrane transport (GO:0034764)	213	5	.29	+	17.26	1.02E-05	6.62E-04
mammary gland development (GO:0030879)	128	3	.17	+	17.24	7.31E-04	2.15E-02
myelination (GO:0042552)	128	3	.17	+	17.24	7.31E-04	2.15E-02
regulation of myeloid leukocyte differentiation (GO:0002761)	128	3	.17	+	17.24	7.31E-04	2.15E-02

regulation of DNA repair (GO:0006282)	214	5	.29	+	17.18	1.04E-05	6.74E-04
positive regulation of protein kinase activity (GO:0045860)	257	6	.35	+	17.17	1.21E-06	1.04E-04
gland development (GO:0048732)	430	10	.58	+	17.10	1.65E-10	5.57E-08
regulation of protein binding (GO:0043393)	129	3	.18	+	17.10	7.47E-04	2.18E-02
regulation of canonical Wnt signaling pathway (GO:0060828)	259	6	.35	+	17.04	1.27E-06	1.08E-04
respiratory system development (GO:0060541)	216	5	.29	+	17.02	1.09E-05	7.02E-04
regulation of macroautophagy (GO:0016241)	173	4	.24	+	17.00	9.15E-05	3.91E-03
positive regulation of DNA metabolic process (GO:0051054)	303	7	.41	+	16.99	1.46E-07	1.68E-05
ensheathment of neurons (GO:0007272)	130	3	.18	+	16.97	7.64E-04	2.21E-02
cellular response to reactive oxygen species (GO:0034614)	130	3	.18	+	16.97	7.64E-04	2.20E-02
axon ensheathment (GO:0008366)	130	3	.18	+	16.97	7.64E-04	2.20E-02
positive regulation of kinase activity (GO:0033674)	304	7	.41	+	16.93	1.50E-07	1.69E-05
negative regulation of kinase activity (GO:0033673)	175	4	.24	+	16.81	9.55E-05	4.07E-03
positive regulation of supramolecular fiber organization (GO:1902905)	175	4	.24	+	16.81	9.55E-05	4.06E-03
digestive tract development (GO:0048565)	133	3	.18	+	16.59	8.15E-04	2.30E-02
regulation of peptidyl-tyrosine phosphorylation (GO:0050730)	222	5	.30	+	16.56	1.24E-05	7.85E-04

positive regulation of lymphocyte differentiation (GO:0045621)	134	3	.18	+	16.46	8.32E -04	2.35E -02
epithelial tube formation (GO:0072175)	134	3	.18	+	16.46	8.32E -04	2.34E -02
negative regulation of epithelial cell proliferation (GO:0050680)	136	3	.18	+	16.22	8.68E -04	2.43E -02
regulation of hemopoiesis (GO:1903706)	409	9	.56	+	16.18	2.61E -09	5.84E -07
branching morphogenesis of an epithelial tube (GO:0048754)	137	3	.19	+	16.10	8.86E -04	2.47E -02
regulation of epithelial cell migration (GO:0010632)	229	5	.31	+	16.06	1.43E -05	8.77E -04
positive regulation of leukocyte cell-cell adhesion (GO:1903039)	276	6	.38	+	15.99	1.81E -06	1.50E -04
positive regulation of lymphocyte activation (GO:0051251)	328	7	.45	+	15.70	2.48E -07	2.62E -05
transmembrane receptor protein tyrosine kinase signaling pathway (GO:0007169)	422	9	.57	+	15.68	3.40E -09	7.21E -07
positive regulation of transferase activity (GO:0051347)	376	8	.51	+	15.65	3.02E -08	4.38E -06
positive regulation of synaptic transmission (GO:0050806)	141	3	.19	+	15.65	9.61E -04	2.65E -02
developmental growth involved in morphogenesis (GO:0060560)	141	3	.19	+	15.65	9.61E -04	2.64E -02
positive regulation of leukocyte differentiation (GO:1902107)	189	4	.26	+	15.56	1.28E -04	5.19E -03

negative regulation of cell growth (GO:0030308)	189	4	.26	+	15.56	1.28E-04	5.18E-03
positive regulation of hemopoiesis (GO:1903708)	189	4	.26	+	15.56	1.28E-04	5.17E-03
regulation of apoptotic signaling pathway (GO:2001233)	381	8	.52	+	15.44	3.34E-08	4.75E-06
regulation of mitotic cell cycle phase transition (GO:1901990)	334	7	.45	+	15.41	2.79E-07	2.90E-05
regulation of binding (GO:0051098)	240	5	.33	+	15.32	1.79E-05	1.05E-03
digestive system development (GO:0055123)	145	3	.20	+	15.22	1.04E-03	2.81E-02
protein-containing complex disassembly (GO:0032984)	146	3	.20	+	15.11	1.06E-03	2.85E-02
positive regulation of chemotaxis (GO:0050921)	146	3	.20	+	15.11	1.06E-03	2.85E-02
tube formation (GO:0035148)	147	3	.20	+	15.01	1.08E-03	2.89E-02
positive regulation of neurogenesis (GO:0050769)	246	5	.33	+	14.95	2.01E-05	1.15E-03
lymphocyte differentiation (GO:0030098)	296	6	.40	+	14.91	2.70E-06	2.12E-04
regulation of protein ubiquitination (GO:0031396)	198	4	.27	+	14.86	1.52E-04	6.01E-03
morphogenesis of embryonic epithelium (GO:0016331)	149	3	.20	+	14.81	1.12E-03	2.97E-02
ameboidal-type cell migration (GO:0001667)	200	4	.27	+	14.71	1.58E-04	6.23E-03
regulation of protein localization to nucleus (GO:1900180)	150	3	.20	+	14.71	1.15E-03	3.01E-02
regulation of mitochondrion organization (GO:0010821)	150	3	.20	+	14.71	1.15E-03	3.00E-02

immune response-activating cell surface receptor signaling pathway (GO:0002429)	250	5	.34	+	14.71	2.17E-05	1.23E-03
regulation of lymphocyte activation (GO:0051249)	502	10	.68	+	14.65	7.17E-10	1.82E-07
placenta development (GO:0001890)	151	3	.21	+	14.61	1.17E-03	3.03E-02
regulation of cold-induced thermogenesis (GO:0120161)	151	3	.21	+	14.61	1.17E-03	3.02E-02
positive regulation of cell development (GO:0010720)	453	9	.62	+	14.61	6.23E-09	1.17E-06
regulation of synaptic plasticity (GO:0048167)	203	4	.28	+	14.49	1.67E-04	6.49E-03
regulation of autophagy (GO:0010506)	357	7	.49	+	14.42	4.34E-07	4.32E-05
positive regulation of leukocyte activation (GO:0002696)	360	7	.49	+	14.30	4.58E-07	4.51E-05
response to UV (GO:0009411)	156	3	.21	+	14.14	1.28E-03	3.22E-02
protein autophosphorylation (GO:0046777)	156	3	.21	+	14.14	1.28E-03	3.22E-02
learning (GO:0007612)	156	3	.21	+	14.14	1.28E-03	3.21E-02
regulation of axonogenesis (GO:0050770)	158	3	.21	+	13.96	1.33E-03	3.32E-02
phagocytosis (GO:0006909)	159	3	.22	+	13.88	1.35E-03	3.35E-02
positive regulation of neuron projection development (GO:0010976)	159	3	.22	+	13.88	1.35E-03	3.34E-02
regulation of leukocyte cell-cell adhesion (GO:1903037)	371	7	.50	+	13.88	5.59E-07	5.39E-05
negative regulation of transferase	214	4	.29	+	13.75	2.04E-04	7.73E-03

activity (GO:0051348)							
hindbrain development (GO:0030902)	161	3	.22	+	13.70	1.40E-03	3.45E-02
positive regulation of cell activation (GO:0050867)	378	7	.51	+	13.62	6.32E-07	5.99E-05
positive regulation of cell-cell adhesion (GO:0022409)	325	6	.44	+	13.58	4.57E-06	3.29E-04
regulation of cell cycle phase transition (GO:1901987)	434	8	.59	+	13.56	8.93E-08	1.13E-05
response to insulin (GO:0032868)	217	4	.30	+	13.56	2.15E-04	8.09E-03
regulation of mitotic cell cycle (GO:0007346)	498	9	.68	+	13.29	1.39E-08	2.34E-06
morphogenesis of a branching epithelium (GO:0061138)	166	3	.23	+	13.29	1.52E-03	3.69E-02
anatomical structure homeostasis (GO:0060249)	224	4	.30	+	13.13	2.42E-04	9.00E-03
tissue homeostasis (GO:0001894)	224	4	.30	+	13.13	2.42E-04	8.97E-03
homeostasis of number of cells (GO:0048872)	282	5	.38	+	13.04	3.81E-05	1.92E-03
mononuclear cell differentiation (GO:1903131)	339	6	.46	+	13.02	5.80E-06	4.11E-04
immune response-regulating cell surface receptor signaling pathway (GO:0002768)	283	5	.38	+	12.99	3.88E-05	1.94E-03
regulation of leukocyte activation (GO:0002694)	566	10	.77	+	12.99	2.23E-09	5.16E-07
regulation of cell activation (GO:0050865)	623	11	.85	+	12.99	2.84E-10	8.18E-08
regulation of cellular response to stress (GO:0080135)	511	9	.69	+	12.95	1.74E-08	2.78E-06
negative regulation of protein	284	5	.39	+	12.95	3.94E-05	1.97E-03

phosphorylation (GO:0001933)							
mesenchymal cell differentiation (GO:0048762)	172	3	.23	+	12.83	1.68E-03	4.05E-02
regulation of DNA metabolic process (GO:0051052)	522	9	.71	+	12.68	2.08E-08	3.27E-06
regulation of protein kinase activity (GO:0045859)	464	8	.63	+	12.68	1.48E-07	1.68E-05
regulation of kinase activity (GO:0043549)	527	9	.72	+	12.56	2.25E-08	3.51E-06
positive regulation of nervous system development (GO:0051962)	294	5	.40	+	12.51	4.63E-05	2.24E-03
morphogenesis of a branching structure (GO:0001763)	177	3	.24	+	12.46	1.83E-03	4.34E-02
response to reactive oxygen species (GO:0000302)	177	3	.24	+	12.46	1.83E-03	4.33E-02
regulation of ERK1 and ERK2 cascade (GO:0070372)	296	5	.40	+	12.42	4.78E-05	2.31E-03
positive regulation of cell projection organization (GO:0031346)	358	6	.49	+	12.33	7.88E-06	5.36E-04
regulation of epithelial cell proliferation (GO:0050678)	361	6	.49	+	12.22	8.25E-06	5.57E-04
regulation of protein modification by small protein conjugation or removal (GO:1903320)	241	4	.33	+	12.21	3.18E-04	1.13E-02
regulation of synapse organization (GO:0050807)	242	4	.33	+	12.16	3.23E-04	1.14E-02
regulation of intrinsic apoptotic signaling pathway (GO:2001242)	182	3	.25	+	12.12	1.97E-03	4.60E-02
positive regulation of cell adhesion (GO:0045785)	486	8	.66	+	12.11	2.09E-07	2.26E-05

negative regulation of phosphorylation (GO:0042326)	304	5	.41	+	12.10	5.42E-05	2.58E-03
negative regulation of growth (GO:0045926)	244	4	.33	+	12.06	3.33E-04	1.16E-02
vasculature development (GO:0001944)	551	9	.75	+	12.01	3.28E-08	4.72E-06
regulation of phosphatidylinositol 3-kinase/protein kinase B signal transduction (GO:0051896)	245	4	.33	+	12.01	3.38E-04	1.18E-02
positive regulation of cytoskeleton organization (GO:0051495)	184	3	.25	+	11.99	2.03E-03	4.74E-02
regulation of post-translational protein modification (GO:1901873)	246	4	.33	+	11.96	3.44E-04	1.19E-02
cell-substrate adhesion (GO:0031589)	185	3	.25	+	11.93	2.07E-03	4.80E-02
cellular response to peptide hormone stimulus (GO:0071375)	248	4	.34	+	11.86	3.54E-04	1.22E-02
regulation of synapse structure or activity (GO:0050803)	248	4	.34	+	11.86	3.54E-04	1.22E-02
positive regulation of phosphorylation (GO:0042327)	622	10	.85	+	11.82	5.43E-09	1.06E-06
response to growth factor (GO:0070848)	506	8	.69	+	11.63	2.83E-07	2.91E-05
response to peptide hormone (GO:0043434)	380	6	.52	+	11.61	1.10E-05	7.05E-04
response to light stimulus (GO:0009416)	318	5	.43	+	11.56	6.68E-05	3.05E-03
positive regulation of cell cycle process (GO:0090068)	255	4	.35	+	11.54	3.93E-04	1.32E-02
regulation of cellular response to growth	319	5	.43	+	11.53	6.78E-05	3.09E-03

factor stimulus (GO:0090287)							
positive regulation of protein phosphorylation (GO:0001934)	576	9	.78	+	11.49	4.78E-08	6.56E-06
cell fate commitment (GO:0045165)	256	4	.35	+	11.49	3.98E-04	1.33E-02
regulation of transferase activity (GO:0051338)	640	10	.87	+	11.49	7.10E-09	1.32E-06
immune response-activating signaling pathway (GO:0002757)	321	5	.44	+	11.46	6.98E-05	3.15E-03
regulation of cell development (GO:0060284)	836	13	1.14	+	11.44	1.88E-11	1.31E-08
T cell activation (GO:0042110)	322	5	.44	+	11.42	7.08E-05	3.19E-03
positive regulation of cell cycle (GO:0045787)	324	5	.44	+	11.35	7.29E-05	3.24E-03
regulation of neurogenesis (GO:0050767)	389	6	.53	+	11.34	1.25E-05	7.93E-04
regulation of transmembrane receptor protein serine/threonine kinase signaling pathway (GO:0090092)	263	4	.36	+	11.19	4.41E-04	1.43E-02
regulation of protein serine/threonine kinase activity (GO:0071900)	264	4	.36	+	11.14	4.47E-04	1.45E-02
blood vessel development (GO:0001568)	530	8	.72	+	11.10	4.00E-07	4.06E-05
regulation of nervous system development (GO:0051960)	464	7	.63	+	11.09	2.42E-06	1.92E-04
regulation of leukocyte proliferation (GO:0070663)	266	4	.36	+	11.06	4.60E-04	1.48E-02

positive regulation of cell differentiation (GO:0045597)	871	13	1.18	+	10.98	3.12E-11	1.76E-08
regulation of actin filament organization (GO:0110053)	271	4	.37	+	10.86	4.92E-04	1.58E-02
multicellular organismal-level homeostasis (GO:0048871)	611	9	.83	+	10.83	7.85E-08	1.01E-05
enzyme-linked receptor protein signaling pathway (GO:0007167)	614	9	.83	+	10.78	8.18E-08	1.05E-05
negative regulation of protein modification process (GO:0031400)	412	6	.56	+	10.71	1.73E-05	1.02E-03
cellular response to chemical stress (GO:0062197)	278	4	.38	+	10.58	5.41E-04	1.71E-02
regulation of cell-cell adhesion (GO:0022407)	487	7	.66	+	10.57	3.32E-06	2.54E-04
positive regulation of phosphorus metabolic process (GO:0010562)	700	10	.95	+	10.51	1.65E-08	2.73E-06
positive regulation of phosphate metabolic process (GO:0045937)	700	10	.95	+	10.51	1.65E-08	2.70E-06
learning or memory (GO:0007611)	280	4	.38	+	10.51	5.56E-04	1.75E-02
regulation of protein phosphorylation (GO:0001932)	913	13	1.24	+	10.47	5.56E-11	2.73E-08
negative regulation of cell development (GO:0010721)	281	4	.38	+	10.47	5.63E-04	1.77E-02
protein phosphorylation (GO:0006468)	492	7	.67	+	10.46	3.55E-06	2.64E-04
regulation of cell growth (GO:0001558)	423	6	.58	+	10.43	2.00E-05	1.15E-03
regulation of phosphorylation (GO:0042325)	989	14	1.34	+	10.41	8.04E-12	7.21E-09

muscle cell differentiation (GO:0042692)	283	4	.38	+	10.39	5.78E-04	1.79E-02
immune response-regulating signaling pathway (GO:0002764)	356	5	.48	+	10.33	1.13E-04	4.69E-03
skin development (GO:0043588)	285	4	.39	+	10.32	5.93E-04	1.82E-02
blood vessel morphogenesis (GO:0048514)	431	6	.59	+	10.24	2.22E-05	1.26E-03
leukocyte differentiation (GO:0002521)	432	6	.59	+	10.21	2.25E-05	1.27E-03
growth (GO:0040007)	432	6	.59	+	10.21	2.25E-05	1.26E-03
developmental growth (GO:0048589)	432	6	.59	+	10.21	2.25E-05	1.26E-03
negative regulation of cell migration (GO:0030336)	288	4	.39	+	10.21	6.17E-04	1.87E-02
positive regulation of organelle organization (GO:0010638)	504	7	.69	+	10.21	4.15E-06	3.04E-04
regulation of cell cycle process (GO:0010564)	726	10	.99	+	10.13	2.31E-08	3.56E-06
negative regulation of phosphate metabolic process (GO:0045936)	365	5	.50	+	10.07	1.27E-04	5.16E-03
cell population proliferation (GO:0008283)	732	10	1.00	+	10.05	2.50E-08	3.81E-06
negative regulation of phosphorus metabolic process (GO:0010563)	366	5	.50	+	10.05	1.28E-04	5.16E-03
negative regulation of cell motility (GO:2000146)	303	4	.41	+	9.71	7.44E-04	2.18E-02
regulation of neuron projection development (GO:0010975)	459	6	.62	+	9.61	3.15E-05	1.67E-03
regulation of actin filament-based	383	5	.52	+	9.60	1.58E-04	6.21E-03

process (GO:0032970)							
embryonic organ development (GO:0048568)	460	6	.63	+	9.59	3.19E-05	1.68E-03
response to peptide (GO:1901652)	462	6	.63	+	9.55	3.26E-05	1.71E-03
cellular response to peptide (GO:1901653)	308	4	.42	+	9.55	7.90E-04	2.26E-02
regulation of small GTPase mediated signal transduction (GO:0051056)	308	4	.42	+	9.55	7.90E-04	2.26E-02
activation of immune response (GO:0002253)	387	5	.53	+	9.50	1.66E-04	6.45E-03
positive regulation of cell migration (GO:0030335)	543	7	.74	+	9.48	6.73E-06	4.68E-04
positive regulation of MAPK cascade (GO:0043410)	467	6	.64	+	9.45	3.46E-05	1.77E-03
cellular response to organonitrogen compound (GO:0071417)	547	7	.74	+	9.41	7.05E-06	4.86E-04
regulation of supramolecular fiber organization (GO:1902903)	391	5	.53	+	9.40	1.74E-04	6.73E-03
regulation of cell adhesion (GO:0030155)	789	10	1.07	+	9.32	5.02E-08	6.77E-06
regulation of organelle organization (GO:0033043)	1185	15	1.61	+	9.31	4.99E-12	5.43E-09
cellular response to growth factor stimulus (GO:0071363)	475	6	.65	+	9.29	3.80E-05	1.92E-03
apoptotic signaling pathway (GO:0097190)	317	4	.43	+	9.28	8.79E-04	2.46E-02
epithelial tube morphogenesis (GO:0060562)	317	4	.43	+	9.28	8.79E-04	2.45E-02
transcription by RNA polymerase II (GO:0006366)	400	5	.54	+	9.19	1.93E-04	7.36E-03

response to lipopolysaccharide (GO:0032496)	321	4	.44	+	9.16	9.20E-04	2.55E-02
cellular response to abiotic stimulus (GO:0071214)	322	4	.44	+	9.14	9.31E-04	2.57E-02
cellular response to environmental stimulus (GO:0104004)	322	4	.44	+	9.14	9.31E-04	2.56E-02
lymphocyte activation (GO:0046649)	487	6	.66	+	9.06	4.36E-05	2.14E-03
cognition (GO:0050890)	325	4	.44	+	9.05	9.63E-04	2.64E-02
regulation of MAPK cascade (GO:0043408)	651	8	.89	+	9.04	1.84E-06	1.52E-04
positive regulation of cell motility (GO:2000147)	570	7	.78	+	9.03	9.20E-06	6.09E-04
regulation of phosphate metabolic process (GO:0019220)	1150	14	1.56	+	8.95	5.88E-11	2.80E-08
regulation of phosphorus metabolic process (GO:0051174)	1151	14	1.57	+	8.95	5.95E-11	2.67E-08
tube development (GO:0035295)	905	11	1.23	+	8.94	1.34E-08	2.27E-06
regulation of cell differentiation (GO:0045595)	1566	19	2.13	+	8.92	2.15E-15	3.27E-11
phosphorylation (GO:0016310)	745	9	1.01	+	8.88	4.12E-07	4.16E-05
positive regulation of locomotion (GO:0040017)	585	7	.80	+	8.80	1.09E-05	6.99E-04
negative regulation of cell differentiation (GO:0045596)	670	8	.91	+	8.78	2.27E-06	1.83E-04
regulation of protein stability (GO:0031647)	335	4	.46	+	8.78	1.08E-03	2.88E-02
regulation of cell cycle (GO:0051726)	1090	13	1.48	+	8.77	4.83E-10	1.29E-07
circulatory system development (GO:0072359)	928	11	1.26	+	8.72	1.73E-08	2.81E-06

negative regulation of locomotion (GO:0040013)	338	4	.46	+	8.70	1.11E-03	2.95E-02
response to molecule of bacterial origin (GO:0002237)	339	4	.46	+	8.68	1.12E-03	2.97E-02
positive regulation of protein modification process (GO:0031401)	763	9	1.04	+	8.67	5.02E-07	4.91E-05
angiogenesis (GO:0001525)	340	4	.46	+	8.65	1.14E-03	2.99E-02
regulation of cell migration (GO:0030334)	940	11	1.28	+	8.61	1.98E-08	3.14E-06
regulation of actin cytoskeleton organization (GO:0032956)	342	4	.47	+	8.60	1.16E-03	3.02E-02
tube morphogenesis (GO:0035239)	684	8	.93	+	8.60	2.65E-06	2.09E-04
response to radiation (GO:0009314)	428	5	.58	+	8.59	2.63E-04	9.60E-03
chemotaxis (GO:0006935)	345	4	.47	+	8.53	1.20E-03	3.09E-02
negative regulation of organelle organization (GO:0010639)	345	4	.47	+	8.53	1.20E-03	3.09E-02
epidermis development (GO:0008544)	346	4	.47	+	8.50	1.21E-03	3.11E-02
taxis (GO:0042330)	347	4	.47	+	8.48	1.22E-03	3.10E-02
regulation of protein modification process (GO:0031399)	1230	14	1.67	+	8.37	1.42E-10	4.93E-08
response to inorganic substance (GO:0010035)	528	6	.72	+	8.36	6.80E-05	3.08E-03
positive regulation of catalytic activity (GO:0043085)	884	10	1.20	+	8.32	1.44E-07	1.66E-05
chromatin remodeling (GO:0006338)	621	7	.84	+	8.29	1.59E-05	9.64E-04
regulation of growth (GO:0040008)	621	7	.84	+	8.29	1.59E-05	9.60E-04
negative regulation of cell population	710	8	.97	+	8.29	3.48E-06	2.60E-04

proliferation (GO:0008285)							
positive regulation of developmental process (GO:0051094)	1337	15	1.82	+	8.25	2.74E-11	1.67E-08
cellular response to nitrogen compound (GO:1901699)	624	7	.85	+	8.25	1.64E-05	9.82E-04
germ cell development (GO:0007281)	362	4	.49	+	8.13	1.43E-03	3.49E-02
response to metal ion (GO:0010038)	363	4	.49	+	8.10	1.44E-03	3.52E-02
regulation of cell motility (GO:2000145)	1000	11	1.36	+	8.09	3.71E-08	5.19E-06
positive regulation of immune system process (GO:0002684)	1000	11	1.36	+	8.09	3.71E-08	5.14E-06
negative regulation of intracellular signal transduction (GO:1902532)	547	6	.74	+	8.07	8.25E-05	3.58E-03
programmed cell death (GO:0012501)	1094	12	1.49	+	8.07	7.31E-09	1.34E-06
regulation of DNA-binding transcription factor activity (GO:0051090)	366	4	.50	+	8.04	1.49E-03	3.62E-02
cell death (GO:0008219)	1098	12	1.49	+	8.04	7.61E-09	1.35E-06
RNA biosynthetic process (GO:0032774)	551	6	.75	+	8.01	8.58E-05	3.71E-03
cell activation (GO:0001775)	736	8	1.00	+	7.99	4.53E-06	3.27E-04
locomotion (GO:0040011)	368	4	.50	+	7.99	1.52E-03	3.68E-02
negative regulation of programmed cell death (GO:0043069)	921	10	1.25	+	7.99	2.10E-07	2.25E-05
intracellular signaling cassette (GO:0141124)	831	9	1.13	+	7.96	1.02E-06	8.91E-05
cellular response to organic cyclic compound (GO:0071407)	466	5	.63	+	7.89	3.87E-04	1.31E-02

synaptic signaling (GO:0099536)	467	5	.64	+	7.87	3.91E -04	1.32E -02
regulation of multicellular organismal development (GO:2000026)	1404	15	1.91	+	7.86	5.45E -11	2.77E -08
chromosome organization (GO:0051276)	468	5	.64	+	7.86	3.95E -04	1.33E -02
regulation of transmembrane transport (GO:0034762)	468	5	.64	+	7.86	3.95E -04	1.32E -02
regulation of plasma membrane bounded cell projection organization (GO:0120035)	656	7	.89	+	7.85	2.26E -05	1.26E -03
positive regulation of molecular function (GO:0044093)	1219	13	1.66	+	7.84	1.87E -09	4.39E -07
cellular process involved in reproduction in multicellular organism (GO:0022412)	469	5	.64	+	7.84	3.99E -04	1.33E -02
heart development (GO:0007507)	566	6	.77	+	7.80	9.94E -05	4.17E -03
regulation of locomotion (GO:0040012)	1042	11	1.42	+	7.76	5.63E -08	7.53E -06
apoptotic process (GO:0006915)	1045	11	1.42	+	7.74	5.79E -08	7.68E -06
positive regulation of cell population proliferation (GO:0008284)	950	10	1.29	+	7.74	2.78E -07	2.91E -05
regulation of cell projection organization (GO:0031344)	672	7	.91	+	7.66	2.63E -05	1.44E -03
positive regulation of transcription by RNA polymerase II (GO:0045944)	1265	13	1.72	+	7.56	2.92E -09	6.37E -07
response to hormone (GO:0009725)	785	8	1.07	+	7.49	7.24E -06	4.97E -04

regulation of cellular catabolic process (GO:0031329)	688	7	.94	+	7.48	3.06E-05	1.64E-03
regulation of cell population proliferation (GO:0042127)	1676	17	2.28	+	7.46	2.90E-12	3.69E-09
positive regulation of protein localization (GO:1903829)	495	5	.67	+	7.43	5.09E-04	1.62E-02
negative regulation of apoptotic process (GO:0043066)	894	9	1.22	+	7.40	1.85E-06	1.52E-04
hemopoiesis (GO:0030097)	699	7	.95	+	7.36	3.38E-05	1.74E-03
response to abiotic stimulus (GO:0009628)	1112	11	1.51	+	7.27	1.08E-07	1.33E-05
cellular response to hormone stimulus (GO:0032870)	506	5	.69	+	7.27	5.62E-04	1.77E-02
in utero embryonic development (GO:0001701)	405	4	.55	+	7.26	2.14E-03	4.91E-02
positive regulation of cellular component organization (GO:0051130)	1118	11	1.52	+	7.24	1.14E-07	1.37E-05
leukocyte activation (GO:0045321)	611	6	.83	+	7.22	1.50E-04	5.95E-03
negative regulation of developmental process (GO:0051093)	919	9	1.25	+	7.20	2.32E-06	1.86E-04
muscle structure development (GO:0061061)	517	5	.70	+	7.11	6.19E-04	1.87E-02
neurogenesis (GO:0022008)	1346	13	1.83	+	7.10	6.16E-09	1.17E-06
regulation of cytoskeleton organization (GO:0051493)	522	5	.71	+	7.04	6.46E-04	1.95E-02
positive regulation of signal transduction (GO:0009967)	1568	15	2.13	+	7.04	2.55E-10	7.79E-08
response to organonitrogen	945	9	1.28	+	7.00	2.91E-06	2.27E-04

compound (GO:0010243)							
regulation of anatomical structure morphogenesis (GO:0022603)	845	8	1.15	+	6.96	1.23E-05	7.87E-04
peptidyl-amino acid modification (GO:0018193)	529	5	.72	+	6.95	6.86E-04	2.03E-02
response to organic cyclic compound (GO:0014070)	848	8	1.15	+	6.94	1.27E-05	7.97E-04
response to lipid (GO:0033993)	852	8	1.16	+	6.91	1.31E-05	8.11E-04
positive regulation of DNA-templated transcription (GO:0045893)	1711	16	2.33	+	6.88	6.31E-11	2.75E-08
positive regulation of RNA biosynthetic process (GO:1902680)	1714	16	2.33	+	6.87	6.48E-11	2.74E-08
epithelial cell differentiation (GO:0030855)	643	6	.87	+	6.86	1.98E-04	7.53E-03
DNA-templated transcription (GO:0006351)	539	5	.73	+	6.82	7.46E-04	2.18E-02
negative regulation of signal transduction (GO:0009968)	1301	12	1.77	+	6.78	4.95E-08	6.73E-06
response to endogenous stimulus (GO:0009719)	1411	13	1.92	+	6.78	1.08E-08	1.87E-06
actin cytoskeleton organization (GO:0030036)	547	5	.74	+	6.72	7.97E-04	2.27E-02
positive regulation of cell communication (GO:0010647)	1754	16	2.39	+	6.71	9.13E-11	3.66E-08
chromatin organization (GO:0006325)	768	7	1.04	+	6.70	6.11E-05	2.82E-03
positive regulation of signaling (GO:0023056)	1756	16	2.39	+	6.70	9.29E-11	3.54E-08
regulation of immune system	1540	14	2.09	+	6.69	2.64E-09	5.83E-07

process (GO:0002682)							
positive regulation of multicellular organismal process (GO:0051240)	1652	15	2.25	+	6.68	5.28E-10	1.36E-07
regulation of response to stress (GO:0080134)	1356	12	1.84	+	6.51	7.78E-08	1.01E-05
head development (GO:0060322)	792	7	1.08	+	6.50	7.41E-05	3.28E-03
positive regulation of nucleobase-containing compound metabolic process (GO:0045935)	2059	18	2.80	+	6.43	5.18E-12	5.26E-09
cellular response to endogenous stimulus (GO:0071495)	1148	10	1.56	+	6.41	1.55E-06	1.31E-04
gamete generation (GO:0007276)	807	7	1.10	+	6.38	8.33E-05	3.61E-03
positive regulation of RNA metabolic process (GO:0051254)	1852	16	2.52	+	6.35	2.05E-10	6.64E-08
positive regulation of intracellular signal transduction (GO:1902533)	1043	9	1.42	+	6.35	6.48E-06	4.53E-04
cell morphogenesis (GO:0000902)	696	6	.95	+	6.34	3.02E-04	1.08E-02
negative regulation of cell communication (GO:0010648)	1397	12	1.90	+	6.32	1.08E-07	1.33E-05
negative regulation of signaling (GO:0023057)	1398	12	1.90	+	6.31	1.08E-07	1.32E-05
response to nitrogen compound (GO:1901698)	1053	9	1.43	+	6.29	7.00E-06	4.85E-04
regulation of programmed cell death (GO:0043067)	1521	13	2.07	+	6.29	2.64E-08	3.98E-06
positive regulation of response to stimulus (GO:0048584)	2242	19	3.05	+	6.23	1.39E-12	1.93E-09

homeostatic process (GO:0042592)	1430	12	1.94	+	6.17	1.39E-07	1.61E-05
embryonic morphogenesis (GO:0048598)	596	5	.81	+	6.17	1.17E-03	3.03E-02
anatomical structure formation involved in morphogenesis (GO:0048646)	971	8	1.32	+	6.06	3.33E-05	1.72E-03
neuron differentiation (GO:0030182)	1095	9	1.49	+	6.04	9.58E-06	6.32E-04
protein-DNA complex organization (GO:0071824)	856	7	1.16	+	6.01	1.20E-04	4.95E-03
regulation of developmental process (GO:0050793)	2448	20	3.33	+	6.01	4.05E-13	8.81E-10
regulation of apoptotic process (GO:0042981)	1476	12	2.01	+	5.98	1.96E-07	2.13E-05
actin filament-based process (GO:0030029)	617	5	.84	+	5.96	1.36E-03	3.36E-02
positive regulation of protein metabolic process (GO:0051247)	1243	10	1.69	+	5.92	3.17E-06	2.44E-04
regulation of cellular localization (GO:0060341)	999	8	1.36	+	5.89	4.08E-05	2.02E-03
cell cycle process (GO:0022402)	880	7	1.20	+	5.85	1.43E-04	5.66E-03
behavior (GO:0007610)	632	5	.86	+	5.82	1.51E-03	3.67E-02
regulation of catalytic activity (GO:0050790)	1408	11	1.91	+	5.75	1.14E-06	9.78E-05
cell migration (GO:0016477)	901	7	1.23	+	5.71	1.65E-04	6.42E-03
cell surface receptor signaling pathway (GO:0007166)	2079	16	2.83	+	5.66	1.13E-09	2.82E-07
generation of neurons (GO:0048699)	1171	9	1.59	+	5.65	1.64E-05	9.83E-04
multicellular organismal	913	7	1.24	+	5.64	1.79E-04	6.90E-03

reproductive process (GO:0048609)							
positive regulation of immune response (GO:0050778)	653	5	.89	+	5.63	1.74E-03	4.17E-02
regulation of immune response (GO:0050776)	917	7	1.25	+	5.61	1.84E-04	7.07E-03
positive regulation of gene expression (GO:0010628)	1181	9	1.61	+	5.60	1.75E-05	1.03E-03
chordate embryonic development (GO:0043009)	664	5	.90	+	5.54	1.87E-03	4.43E-02
nucleobase-containing compound biosynthetic process (GO:0034654)	938	7	1.28	+	5.49	2.11E-04	7.98E-03
negative regulation of protein metabolic process (GO:0051248)	810	6	1.10	+	5.45	6.73E-04	2.00E-02
cell motility (GO:0048870)	1081	8	1.47	+	5.44	7.10E-05	3.19E-03
regulation of cellular component organization (GO:0051128)	2442	18	3.32	+	5.42	9.06E-11	3.73E-08
positive regulation of nitrogen compound metabolic process (GO:0051173)	2998	22	4.08	+	5.40	6.35E-14	3.23E-10
regulation of intracellular signal transduction (GO:1902531)	1781	13	2.42	+	5.37	1.69E-07	1.88E-05
multicellular organism reproduction (GO:0032504)	959	7	1.30	+	5.37	2.41E-04	9.00E-03
embryo development ending in birth or egg hatching (GO:0009792)	685	5	.93	+	5.37	2.14E-03	4.91E-02
regulation of response to external stimulus (GO:0032101)	1098	8	1.49	+	5.36	7.92E-05	3.48E-03

regulation of signal transduction (GO:0009966)	3021	22	4.11	+	5.36	7.46E-14	2.27E-10
negative regulation of gene expression (GO:0010629)	968	7	1.32	+	5.32	2.56E-04	9.36E-03
negative regulation of response to stimulus (GO:0048585)	1664	12	2.26	+	5.30	7.09E-07	6.63E-05
regulation of cellular component biogenesis (GO:0044087)	974	7	1.32	+	5.29	2.65E-04	9.65E-03
cell development (GO:0048468)	2240	16	3.05	+	5.25	3.36E-09	7.21E-07
cell cycle (GO:0007049)	1265	9	1.72	+	5.23	3.02E-05	1.63E-03
positive regulation of cellular biosynthetic process (GO:0031328)	2686	19	3.65	+	5.20	3.46E-11	1.88E-08
positive regulation of biosynthetic process (GO:0009891)	2707	19	3.68	+	5.16	3.97E-11	2.09E-08
positive regulation of transport (GO:0051050)	855	6	1.16	+	5.16	8.91E-04	2.48E-02
developmental process involved in reproduction (GO:0003006)	1000	7	1.36	+	5.15	3.11E-04	1.11E-02
central nervous system development (GO:0007417)	1008	7	1.37	+	5.11	3.27E-04	1.15E-02
positive regulation of macromolecule biosynthetic process (GO:0010557)	2604	18	3.54	+	5.08	2.64E-10	7.73E-08
heterocycle biosynthetic process (GO:0018130)	1014	7	1.38	+	5.08	3.39E-04	1.18E-02
positive regulation of cellular metabolic process (GO:0031325)	3341	23	4.54	+	5.06	3.07E-14	2.34E-10
regulation of catabolic process (GO:0009894)	1022	7	1.39	+	5.04	3.55E-04	1.22E-02

aromatic compound biosynthetic process (GO:0019438)	1024	7	1.39	+	5.03	3.59E-04	1.23E-02
regulation of transport (GO:0051049)	1617	11	2.20	+	5.00	4.37E-06	3.19E-04
nervous system development (GO:0007399)	2207	15	3.00	+	5.00	2.79E-08	4.17E-06
regulation of multicellular organismal process (GO:0051239)	2966	20	4.03	+	4.96	1.49E-11	1.19E-08
regulation of protein localization (GO:0032880)	891	6	1.21	+	4.95	1.10E-03	2.93E-02
anatomical structure morphogenesis (GO:0009653)	2239	15	3.04	+	4.93	3.39E-08	4.79E-06
sexual reproduction (GO:0019953)	1052	7	1.43	+	4.89	4.23E-04	1.39E-02
cellular response to oxygen-containing compound (GO:1901701)	1055	7	1.43	+	4.88	4.30E-04	1.40E-02
embryo development (GO:0009790)	1059	7	1.44	+	4.86	4.40E-04	1.43E-02
intracellular signal transduction (GO:0035556)	1514	10	2.06	+	4.86	1.80E-05	1.06E-03
regulation of protein metabolic process (GO:0051246)	2145	14	2.92	+	4.80	1.77E-07	1.94E-05
regulation of molecular function (GO:0065009)	2008	13	2.73	+	4.76	6.75E-07	6.35E-05
positive regulation of macromolecule metabolic process (GO:0010604)	3413	22	4.64	+	4.74	9.52E-13	1.81E-09
cell-cell signaling (GO:0007267)	1086	7	1.48	+	4.74	5.11E-04	1.63E-02
response to oxygen-containing compound (GO:1901700)	1554	10	2.11	+	4.73	2.26E-05	1.26E-03
regulation of localization (GO:0032879)	2028	13	2.76	+	4.71	7.56E-07	6.94E-05

regulation of signaling (GO:0023051)	3434	22	4.67	+	4.71	1.08E-12	1.83E-09
regulation of cell communication (GO:0010646)	3440	22	4.68	+	4.70	1.12E-12	1.71E-09
epithelium development (GO:0060429)	1111	7	1.51	+	4.63	5.86E-04	1.80E-02
cellular response to stress (GO:0033554)	1588	10	2.16	+	4.63	2.72E-05	1.48E-03
positive regulation of metabolic process (GO:0009893)	3719	23	5.06	+	4.55	3.23E-13	8.20E-10
regulation of transcription by RNA polymerase II (GO:0006357)	2603	16	3.54	+	4.52	2.95E-08	4.36E-06
organic cyclic compound biosynthetic process (GO:1901362)	1150	7	1.56	+	4.48	7.19E-04	2.12E-02
response to organic substance (GO:0010033)	2467	15	3.35	+	4.47	1.24E-07	1.47E-05
regulation of response to stimulus (GO:0048583)	3982	24	5.41	+	4.43	7.12E-14	2.71E-10
animal organ morphogenesis (GO:0009887)	996	6	1.35	+	4.43	1.95E-03	4.54E-02
phosphate-containing compound metabolic process (GO:0006796)	1711	10	2.33	+	4.30	5.16E-05	2.47E-03
negative regulation of cellular metabolic process (GO:0031324)	2572	15	3.50	+	4.29	2.16E-07	2.31E-05
regulation of DNA-templated transcription (GO:0006355)	3440	20	4.68	+	4.28	2.33E-10	7.25E-08
cellular response to organic substance (GO:0071310)	1725	10	2.35	+	4.26	5.53E-05	2.61E-03
regulation of RNA biosynthetic process (GO:2001141)	3459	20	4.70	+	4.25	2.58E-10	7.71E-08

phosphorus metabolic process (GO:0006793)	1737	10	2.36	+	4.23	5.87E-05	2.74E-03
negative regulation of metabolic process (GO:0009892)	2828	16	3.85	+	4.16	9.61E-08	1.21E-05
tissue development (GO:0009888)	1769	10	2.41	+	4.16	6.85E-05	3.10E-03
immune system process (GO:0002376)	2316	13	3.15	+	4.13	3.41E-06	2.57E-04
cytoskeleton organization (GO:0007010)	1255	7	1.71	+	4.10	1.20E-03	3.09E-02
animal organ development (GO:0048513)	2869	16	3.90	+	4.10	1.18E-07	1.40E-05
reproductive process (GO:0022414)	1470	8	2.00	+	4.00	5.81E-04	1.79E-02
reproduction (GO:0000003)	1482	8	2.02	+	3.97	6.13E-04	1.86E-02
negative regulation of macromolecule metabolic process (GO:0010605)	2609	14	3.55	+	3.95	1.96E-06	1.60E-04
system development (GO:0048731)	3546	19	4.82	+	3.94	4.40E-09	8.94E-07
negative regulation of macromolecule biosynthetic process (GO:0010558)	2061	11	2.80	+	3.93	4.36E-05	2.14E-03
regulation of RNA metabolic process (GO:0051252)	3758	20	5.11	+	3.91	1.18E-09	2.90E-07
negative regulation of cellular biosynthetic process (GO:0031327)	2113	11	2.87	+	3.83	5.48E-05	2.60E-03
cell differentiation (GO:0030154)	3654	19	4.97	+	3.82	7.36E-09	1.34E-06
cellular nitrogen compound biosynthetic process (GO:0044271)	1539	8	2.09	+	3.82	7.86E-04	2.26E-02
cellular developmental process (GO:0048869)	3657	19	4.97	+	3.82	7.46E-09	1.34E-06
negative regulation of biosynthetic	2127	11	2.89	+	3.80	5.83E-05	2.73E-03

process (GO:0009890)							
cellular response to chemical stimulus (GO:0070887)	2389	12	3.25	+	3.69	3.05E-05	1.64E-03
regulation of nucleobase-containing compound metabolic process (GO:0019219)	4061	20	5.52	+	3.62	4.84E-09	9.70E-07
protein modification process (GO:0036211)	2069	10	2.81	+	3.55	2.53E-04	9.29E-03
response to external stimulus (GO:0009605)	2290	11	3.11	+	3.53	1.15E-04	4.74E-03
multicellular organism development (GO:0007275)	3964	19	5.39	+	3.53	2.95E-08	4.32E-06
response to chemical (GO:0042221)	3780	18	5.14	+	3.50	1.13E-07	1.36E-05
regulation of gene expression (GO:0010468)	4898	23	6.66	+	3.45	1.27E-10	4.49E-08
regulation of cellular biosynthetic process (GO:0031326)	5159	24	7.01	+	3.42	2.65E-11	1.68E-08
protein localization (GO:0008104)	1944	9	2.64	+	3.40	7.84E-04	2.26E-02
regulation of biosynthetic process (GO:0009889)	5190	24	7.06	+	3.40	3.04E-11	1.78E-08
cellular macromolecule localization (GO:0070727)	1951	9	2.65	+	3.39	8.05E-04	2.29E-02
nucleic acid metabolic process (GO:0090304)	2180	10	2.96	+	3.37	3.87E-04	1.31E-02
regulation of macromolecule biosynthetic process (GO:0010556)	5026	23	6.83	+	3.37	2.20E-10	7.00E-08
negative regulation of nitrogen compound metabolic process (GO:0051172)	2188	10	2.98	+	3.36	3.99E-04	1.33E-02

regulation of nitrogen compound metabolic process (GO:0051171)	5474	25	7.44	+	3.36	5.80E-12	5.53E-09
regulation of biological quality (GO:0065008)	2849	13	3.87	+	3.36	3.34E-05	1.72E-03
negative regulation of cellular process (GO:0048523)	4848	22	6.59	+	3.34	1.28E-09	3.10E-07
regulation of primary metabolic process (GO:0080090)	5622	25	7.64	+	3.27	1.10E-11	9.29E-09
protein-containing complex organization (GO:0043933)	2027	9	2.76	+	3.27	1.06E-03	2.86E-02
anatomical structure development (GO:0048856)	5231	23	7.11	+	3.23	5.18E-10	1.36E-07
response to stress (GO:0006950)	3419	15	4.65	+	3.23	8.61E-06	5.78E-04
positive regulation of cellular process (GO:0048522)	5707	25	7.76	+	3.22	1.57E-11	1.14E-08
macromolecule modification (GO:0043412)	2291	10	3.12	+	3.21	5.77E-04	1.79E-02
regulation of cellular metabolic process (GO:0031323)	6054	26	8.23	+	3.16	3.03E-12	3.56E-09
negative regulation of biological process (GO:0048519)	5208	22	7.08	+	3.11	5.42E-09	1.07E-06
organelle organization (GO:0006996)	3099	13	4.21	+	3.09	8.21E-05	3.58E-03
regulation of macromolecule metabolic process (GO:0060255)	6033	25	8.20	+	3.05	5.89E-11	2.72E-08
positive regulation of biological process (GO:0048518)	6210	25	8.44	+	2.96	1.17E-10	4.25E-08
nucleobase-containing compound metabolic process (GO:0006139)	2743	11	3.73	+	2.95	5.68E-04	1.76E-02

developmental process (GO:0032502)	5739	23	7.80	+	2.95	3.71E-09	7.75E-07
regulation of metabolic process (GO:0019222)	6559	26	8.92	+	2.92	2.27E-11	1.50E-08
heterocycle metabolic process (GO:0046483)	2931	11	3.99	+	2.76	1.00E-03	2.74E-02
cellular aromatic compound metabolic process (GO:0006725)	2971	11	4.04	+	2.72	1.13E-03	2.97E-02
cellular nitrogen compound metabolic process (GO:0034641)	3288	12	4.47	+	2.68	6.75E-04	2.01E-02
multicellular organismal process (GO:0032501)	6745	24	9.17	+	2.62	1.08E-08	1.85E-06
signal transduction (GO:0007165)	4789	17	6.51	+	2.61	2.47E-05	1.36E-03
cellular component organization (GO:0016043)	5636	20	7.66	+	2.61	1.59E-06	1.33E-04
cellular component organization or biogenesis (GO:0071840)	5852	20	7.96	+	2.51	3.02E-06	2.34E-04
signaling (GO:0023052)	5109	17	6.95	+	2.45	6.01E-05	2.79E-03
cell communication (GO:0007154)	5262	17	7.16	+	2.38	8.98E-05	3.84E-03
cellular response to stimulus (GO:0051716)	6448	20	8.77	+	2.28	1.54E-05	9.38E-04
response to stimulus (GO:0050896)	8182	25	11.13	+	2.25	7.53E-08	9.90E-06
macromolecule metabolic process (GO:0043170)	5648	16	7.68	+	2.08	1.03E-03	2.79E-02
cellular metabolic process (GO:0044237)	6416	18	8.72	+	2.06	3.43E-04	1.19E-02
metabolic process (GO:0008152)	7979	21	10.85	+	1.94	1.28E-04	5.17E-03
regulation of cellular process (GO:0050794)	11167	27	15.18	+	1.78	1.07E-06	9.35E-05

regulation of biological process (GO:0050789)	11722	27	15.94	+	1.69	3.62E-06	2.68E-04
biological regulation (GO:0065007)	12141	27	16.51	+	1.64	9.19E-06	6.11E-04
